# Integrating LC-MS and HS-GC-MS for the metabolite characterization of the Chinese medicinal plant *Platostoma palustre* under different processing methods

**DOI:** 10.3389/fnut.2023.1181942

**Published:** 2023-05-19

**Authors:** Danfeng Tang, Changqian Quan, Suhua Huang, Fan Wei

**Affiliations:** ^1^Guangxi Key Laboratory of Medicinal Resources Protection and Genetic Improvement, Guangxi Botanical Garden of Medicinal Plants, Nanning, China; ^2^National Traditional Chinese Medicine Inheritance and Innovation Center, Guangxi Botanical Garden of Medicinal Plants, Nanning, China; ^3^College of Pharmacy, Guangxi Medical University, Nanning, China

**Keywords:** *Platostoma palustre*, processing methods, LC-MS, HS-GC-MS, metabolites, volatile substances

## Abstract

*Platostoma palustre* (or *Mesona chinensis* Benth) is an important medicinal and edible plant in China and Southeast Asian countries. To study the effects of different processing methods on the quality, nutrition, and flavor of *P. palustre*, we adopted the LC-MS and HS-GC-MS to compare the influences of tedding (S), sweating (M), and drying (H) on the metabolites and volatile substances of *P. palustre*. Biochemical determinations revealed that the M treatment could promote the accumulation of the contents of total sugar, soluble sugar, and total pectin compared with the H and S treatments but decrease the total flavonoid contents. LC-MS and HS-GC-MS uncovered 98 differential metabolites and 27 differential volatile substances among the three treatments, respectively. Overall, the M treatment facilitated the stabilization and improvement of the quality of polysaccharides and volatile substances, while the H treatment could promote the level of amino acids in *P. palustre*. The current study provided a theoretical reference for establishing standardized processing methods and sustaining the quality stability of *P. palustre* in future.

## 1. Introduction

*Platostoma palustre* (or *Mesona chinensis* Benth), also known as “Xiancao,” is an annual herb of the Labiatae family, which is distributed in the provinces of Guangxi, Guangdong, Taiwan, Zhejiang, and Jiangxi in China (1). *P. palustre* is an important medicinal and edible plant (2, 3), with the above-ground part or the whole plant constituting its medicinal parts. According to the records of “Chinese Materia Medica” and “Dictionary of Traditional Chinese Medicine,” *P. palustre* is effective in treating thirst and hypertension (4, 5). Modern research reports that *P. palustre* possesses antioxidation (6), antihypertensive (7), antibiosis (8), and hypolipidemic effects (9). *P. palustre* is rich in polysaccharides, flavonoids, phenolic acid, volatile oil, and other components (10). Among those, polysaccharides (or xiancao gum) represent one of the most important quality indicators of *P. palustre* (11), and volatile oil endows the species with a special flavor quality (12). At present, *P. palustre* is mainly used to produce herbal tea and jelly. With the development of the herbal tea industry, new products made of *P. palustre* are becoming increasingly popular, thus gradually making it a research hotspot (13).

Generally, the quality of traditional Chinese medicine (TCM) is affected by many factors, such as the variety, origin, harvesting, storage, processing methods, and extraction of the effective components of TCM. Among these, the processing methods include pretreatment, processing, and pulverization of TCM (14). Sweating (micro-fermentation) treatment is the primary processing method for many TCM. *Magnolia officinalis* has a brown surface, and its cross-section turns purplish-red after sweating treatment and the content of magnolol increases while the content of volatile oil and alkaloids decreases (15). The sweating treatment of *Poria cocos* after traditional sweating is whiter with better lustre compared with that after steaming, improving the water solubility of its polysaccharides (16). Tedding refers to use the heat emitted by the sun to evaporate water from medicinal materials, while the traditional drying method involves setting up a simple drying room, using outdoor auxiliary equipment to heat the room, and an exhaust fan to extract the generated moisture outside. Both of these methods are necessary steps for the primary processing of many TCM.

In fact, sweating treatment is also the primary processing method in the *P. palustre* industry. The whole herb medicine of *P. palustre* is usually harvested before flowering. After harvesting and proper drying, the sweating treatment should be carried out in the production area. Subsequently, through the sweating treatment by stacking, the final herbal medicine of *P. palustre* would be brownish or brown in color, having a unique aroma (17). Lu (18) reported that there were some differences in the total flavonoids and total sugar content of *Mesina chinensis Benth* with different (micro-fermentation, shade drying, and oven drying) processing methods, and the content of total flavonoids in *M. chinensis* treated by micro-fermentation was found to be lower than that in the dried samples. However, there was no obvious difference between the volatile components of the sample of micro-fermentation and the dry samples, but after fermentation was performed two times, the relative content of caryophyllene in the samples decreased significantly (β-caryophyllene 0.85% and α-caryophyllene 0.37%), while fora-selinene and β-selinene had little effect (12, 18).

Up to now, there have been few comprehensive reports on the effects of processing methods on the metabolites and volatile substances of *P. palustre*. Therefore, in this study, we compared and analyzed the effects of different (tedding, drying, and sweating) processing treatments on the metabolites and volatile substances of *P. palustre*. Here, we adopted the LC-MS and HS-GC-MS methods to compare the effects of tedding, sweating, and drying on the quality and flavor of *P. palustre*, thus providing a theoretical reference for establishing standardized processing methods and maintaining the quality stability of *P. palustre* in future.

## 2. Materials and methods

### 2.1. Sample preparation

**Figure 1:**
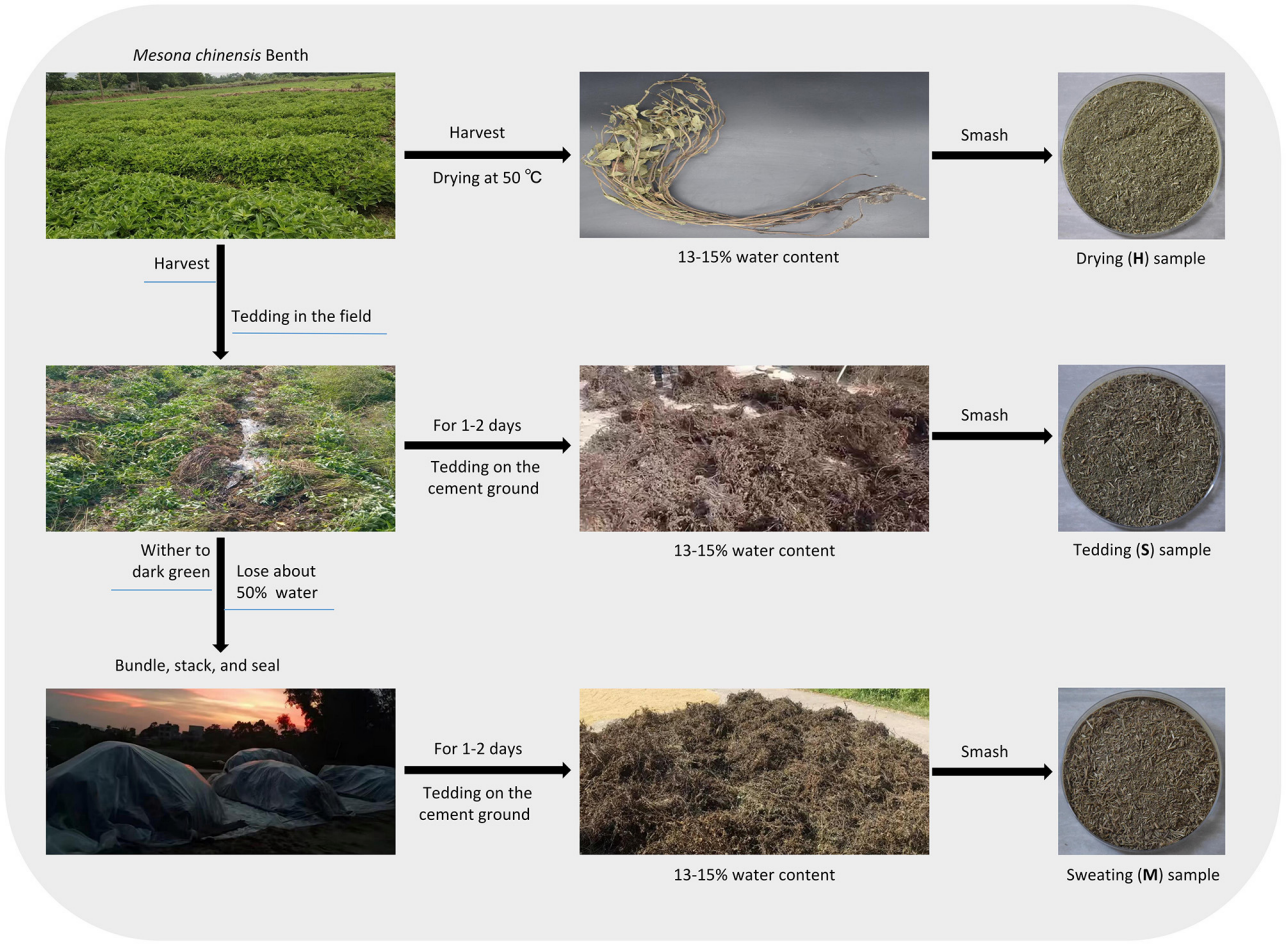
Schematic diagram of the experimental treatment flow in this study.

The *P. palustre* plants from the same variety (Lingshan) and fields were harvested on 25 July 2022. Subsequently, the fresh plant materials were treated with sweating (M), tedding (S), and drying (H), respectively (Figure 1). Sweating (M) method: Collect the aerial part of *P. palustre*, remove the roots and redundant soil, and spread and ted the materials in the field. When the materials wither to dark green and lose ~½ of their water content, bundle, stack, and seal the materials with transparent plastic film on the cement ground. Soak the package in the sun for 1 or 2 days, remove the film, spread out, and dry the materials until the stems are fragile, the leaves are slightly flexible, and the water content is ~13–15%. Tedding (S) method: The initial procedure is the same as above. When the materials wither to a dark green color and lose half of their water, transfer them to a cement ground to continue tedding (turn it over every 2 h) until the stems are fragile, the leaves are slightly flexible, and the water content is ~13–15%. Cover them with plastic film in the evening to prevent rain. Drying (H) method: Place the fresh plant materials in the oven and dry at a low temperature of 50°C until the moisture content is ~13–15%. Cut the processed materials into small sections, then grind them into powder, and sieved them with a 200 mesh sieve. All samples were used for subsequent analysis.

### 2.2. Biochemical determination

The contents of total sugar, soluble sugar, total pectin, and total flavonoid were detected using the total sugar content kit (G0503F), soluble sugar content kit (G0501F), total pectin content kit (G0717F), and total flavonoid kit (G0118F), respectively (Grace Biotechnology, http://geruisi-bio.com/, Suzhou, China). All the kits were measured using a spectrophotometer method.

### 2.3. UHPLC-MS/MS analysis

A total of six biological replicates were used per treatment. The metabolite extraction was utilized for LC/MS analysis (19), with minor adjustments, and a bioinformatic analysis was carried out at Shanghai Majorbio Co., Ltd. (http://wwwmajorbiocom/) following standard procedures.

#### 2.3.1. Metabolite extraction

In total, 50 mg of powder samples were accurately weighed, and the metabolites were extracted using a 400 μL methanol: water (4:1, v/v) solution with 0.02 mg/mL L-2-chlorophenylalanin as an internal standard. The mixture was allowed to settle at −10°C, treated by high-throughput tissue crusher Wonbio-96c (Shanghai Wanbo Biotechnology Co., LTD) at 50 Hz for 6 min, and then followed by ultrasound at 40 kHz for 30 min at 5°C. The samples were placed at −20°C for 30 min to precipitate proteins. After centrifugation at 13,000 × *g* at 4°C for 15 min, the supernatant was carefully transferred to sample vials for LC-MS/MS (UHPLC-Q Exactive HF-X system, Thermo Fisher Scientific) analysis.

#### 2.3.2. Quality control sample

As a part of the system conditioning and quality control process, a pooled quality control (QC) sample was prepared by mixing equal volumes of all samples. The QC samples were disposed of and tested in the same manner as the analytic samples, thereby helping to represent the whole sample set, which was injected at regular intervals (every 5–15 samples), in order to monitor the stability of the analysis.

#### 2.3.3. Chromatographic conditions

In total, 2 μL of the sample was separated by an HSS T3 column (100 mm × 2.1 mm i.d., 1.8 μm) and then entered into mass spectrometry detection. The mobile phases consisted of 0.1% formic acid in water:acetonitrile (95:5, v/v; solvent A) and 0.1% formic acid in acetonitrile:isopropanol:water (47.5:47.5:5, v/v; solvent B). The solvent gradient changed according to the following conditions: from 0 to 3.5 min, 0% B to 24.5% B (0.4 mL/min); from 3.5 to 5 min, 24.5% B to 65% B (0.4 mL/min); from 5 to 5.5 min, 65% B to 100% B (0.4 mL/min); from 5.5 to 7.4 min, 100% B to 100% B (0.4 to 0.6 mL/min); from 7.4 to 7.6 min, 100% B to 51.5% B (0.6 mL/min); from 7.6 to 7.8 min, 51.5% B to 0% B (0.6 to 0.5 mL/min); from 7.8 to 9 min, 0% B to 0% B (0.5 to 0.4 mL/min); and from 9 to 10 min, 0% B to 0% B (0.4 mL/min) for equilibrating the systems. The sample injection volume was 2 μL, and the flow rate was set to 0.4 mL/min, with the column temperature maintained at 40°C. During the period of analysis, all these samples were stored at 4°C.

#### 2.3.4. MS conditions

The mass spectrometric data were collected using a Thermo UHPLC -Q Exactive HF-X Mass Spectrometer, equipped with an electrospray ionization (ESI) source operating in either positive or negative ion modes. The optimal conditions were set as followed: heater temperature, 425°C; capillary temperature, 325°C; sheath gas flow rate, 50 arb; aux gas flow rate, 13 arb; ion-spray voltage floating (ISVF), −3,500 V in negative mode, and 3,500 V in positive mode, respectively. Normalized collision energy and 20-40-60V rolling were employed for MS/MS. Full MS resolution was 60,000, and MS/MS resolution was 7,500. Data acquisition was performed using the Data Dependent Acquisition (DDA) mode. The detection was carried out over a mass range of 70–1,050 m/z.

#### 2.3.5. Data preprocessing and annotation

After the mass spectrometry detection was completed, the raw data of LC/MS were preprocessed by Progenesis QI software (Waters Corporation, Milford, USA), and a three-dimensional data matrix in CSV format was exported. The information in this three-dimensional matrix included as follows: sample information, metabolite name, and mass spectral response intensity. Internal standard peaks and any known false positive peaks (including noise, column bleed, and derivatized reagent peaks) were removed from the data matrix, deredundant, and peak pooled. At the same time, the metabolites were searched and identified, with the main databases being the HMDB (http://www.hmdb.ca/), Metlin (https://metlin.scripps.edu/), and Majorbio databases.

The data obtained after the database search were uploaded to the Majorbio cloud platform (https://cloud.majorbio.com) for further analysis. Metabolic features detected in at least 80% of any set of samples were retained. After filtering, minimum metabolite values were imputed for specific samples, in which the metabolite levels fell below the lower limit of quantitation, and each metabolic feature was normalized by sum normalization. To reduce the errors caused by sample preparation and instrument instability, the response intensity of the sample mass spectrum peaks was normalized by the sum normalization method, thereby obtaining the normalized data matrix. At the same time, variables with relative standard deviation (RSD) > 30% of QC samples were removed, and log10 logarithmic transformation was performed to obtain the final data matrix for subsequent analysis.

#### 2.3.6. Differential metabolites analysis

Variance analysis was performed on the matrix file after data preprocessing. The R package ropls (version 1.6.2) was used to perform principal component analysis (PCA) and orthogonal least partial squares discriminant analysis (OPLS-DA), and a seven-cycle interactive validation was used to evaluate the stability of the model. In addition, the Student's *t*-test and fold difference analysis were carried out. The selection of significantly different metabolites was determined based on the variable importance in the projection (VIP) obtained by the OPLS-DA model and the *p*-value of the Student's *t*-test, and the metabolites with VIP > 1 and *p* < 0.05 were significantly different metabolites (20).

Differential metabolites among the two groups were summarized and mapped into their biochemical pathways through metabolic enrichment and pathway analysis based on a database search (KEGG, http://www.genome.jp/kegg/). Scipy.stats (Python packages; https://docs.scipy.org/doc/scipy/) was exploited to identify statistically significantly enriched pathways using Fisher's exact test.

### 2.4. HS-GC-MS analysis

In this study, six biological replicates were also employed per treatment. The extraction of volatile substances was used for HS-GC-MS analysis as per the method of Dong et al. (21), with minor modifications, and a bioinformatic analysis was carried out at Shanghai Majorbio Co., Ltd. (http://wwwmajorbiocom/), followed by standard procedures.

#### 2.4.1. Extraction

Accurately, 3 g of the sample was weighed and placed into a 20 ml headspace bottle, and then the headspace bottle was immediately sealed until the analysis.

#### 2.4.2. HS-GC-MS analysis

The analysis was performed using an Agilent 8890B gas chromatography, with a 7697A headspace sampler coupled to an Agilent 7000D mass selective detector with an inert electron impact (EI) ionization source and ionization voltage was 70 eV (Agilent, USA). Analyte compounds were separated with a VF-WAXms (25 m × 0.25 mm × 0.2 μm) capillary column, using 99.999% helium as a carrier gas at a constant flow rate (1 mL/ min). The temperature of the headspace strip heating box was 130°C, the quantitative ring was 150°C, the transmission line was 170°C, the balance time of the sample bottle was 20 min, and the GC cycle time was 35 min. The GC column temperature was programmed to hold at 40°C for 2 min and rose to 100°C at a rate of 5°C per minute, then rose to 230°C at a rate of 15°C per minute, for 5 min. The injection volume of samples was 1 μL and introduced in splitting mode (10:1) with the inlet temperature of 180°C. The ion sources temperature was 230°C, and the quadrupole temperature was 150°C. The scanning mode was full scan mode, the quality scanning range was 30–1,000 m/z, and the scanning frequency was 3.2 scan/s.

#### 2.4.3. Quality control

To evaluate the stability of the analytical system during the run-on process, a quality control (QC) sample was prepared during the experiment. QC samples were prepared by mixing all test samples and were handled in the same way as the formal samples. During instrument testing, a QC sample was inserted every 5–15 samples. The repeatability of QC samples reflects the stability of the instrument in the whole analysis process. At the same time, it can also be used to identify variables with significant variations in the analysis system to ensure the reliability of the results.

#### 2.4.4. Data preprocessing and annotation

After the mass spectrometry detection was completed, the raw data of GC/MS were preprocessed by MassHunter Workstation Quantitative Analysis (v10.0.707.0) software, and a three-dimensional data matrix in CSV format was exported. The internal standard peaks and any known false positive peaks were removed from the data matrix, deredundant, and peak pooled. Meanwhile, the metabolites were searched and identified, and the main database used was the Fiehn and NIST public databases.

The data after being retrieved from the database search were uploaded to the Majorbio cloud platform (https://cloud.majorbio.com) for analysis. Metabolic features detected at least 80% in any set of samples were retained. After filtering, minimum metabolite values were imputed for specific samples, in which the metabolite levels fell below the lower limit of quantitation, and each metabolic feature was normalized by sum. To reduce the errors caused by sample preparation and instrument instability, the response intensity of the sample mass spectrum peaks was normalized by the sum normalization method, thus obtaining a normalized data matrix. At the same time, variables with relative standard deviation (RSD) > 30% of QC samples were removed, and log10 logarithmization was performed to obtain the final data matrix for subsequent analysis.

#### 2.4.5. Differential metabolites analysis

Variance analysis was conducted on the matrix file after data preprocessing. The R package ropls (version 1.6.2) was used to perform principal component analysis (PCA) and orthogonal least partial squares discriminant analysis (OPLS-DA), and a seven-cycle interactive validation was used to evaluate the stability of the model. In addition, Student's *t*-test and fold difference analysis were performed. The selection of significantly different metabolites was determined based on the variable importance in the projection (VIP), obtained by the OPLS-DA model and the *p*-value of Student's *t*-test, and the metabolites with VIP > 1 and *p* < 0.05 were considered to be significantly different metabolites (20).

### 2.5. Statistical analysis

SPSS 17.0 software was employed for statistical analysis, and the data means were analyzed using the Duncan test for statistical significance (*p* < 0.05). Each test was analyzed independently, and the test for homogeneity of variance was performed prior to ANOVA. GraphPad Prism 7 and WPS software were used for data processing and graphic analysis.

## 3. Results

### 3.1. Influence of different processing methods on the quality of *P. palustre*

**Figure 2:**
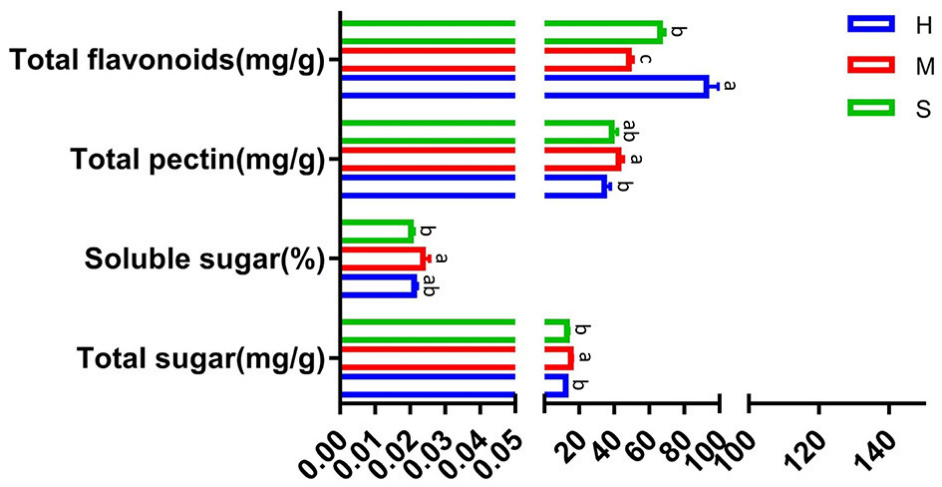
Comparison of total sugar, soluble sugar, total pectin, and total flavonoids under different treatments. The treatments containing the same letters indicated no significant differences (*p* > 0.05), while the treatments containing different letters indicated significant differences (*p* < 0.05).

As shown in Figure 2, compared with the sweating (M) treatment, the total sugar contents significantly decreased by 19.9 and 14.5% in the drying (H) and tedding (S) treatments, respectively. Meanwhile, the soluble sugar contents dramatically reduced by 10.4 and 14.2%, and the total pectin contents significantly decreased by 18.9 and 8.9% in H and S treatments, respectively. However, the total flavonoid contents in H and S treatments significantly increased by 91.2 and 36.5% in comparison with the M treatment.

### 3.2. Metabolites of different treatments based on LC-MS

In this study, the metabolites were extracted from the aerial part of *P. palustre* plants, with six replicates of each treatment, and analyzed based on LC-MS. A total of 1,591 and 1,595 metabolites were identified under the positive and negative ion scanning modes, respectively (Supplementary Figure 1). Analysis of correlation, PCA, Venn, and PLS-DA showed that the data were reliable (Supplementary Figure 2). Based on these metabolites, the Kyoto Encyclopedia of Genes and Genomes (KEGG) compound classification revealed that a total of 174 metabolites could be categorized as phospholipids, monosaccharides, fatty acids, carboxylic acids, eicosanoids, amino acids, and vitamins (Supplementary Figure 3A; Supplementary Tables 1, 2). Further analysis of the KEGG pathway showed that they were involved in lipid metabolism, carbohydrate metabolism, biosynthesis of other secondary metabolites, amino acid metabolism, and metabolism of cofactors and vitamins (Supplementary Figure 3B).

### 3.3. Differential metabolites analysis

**Figure 3:**
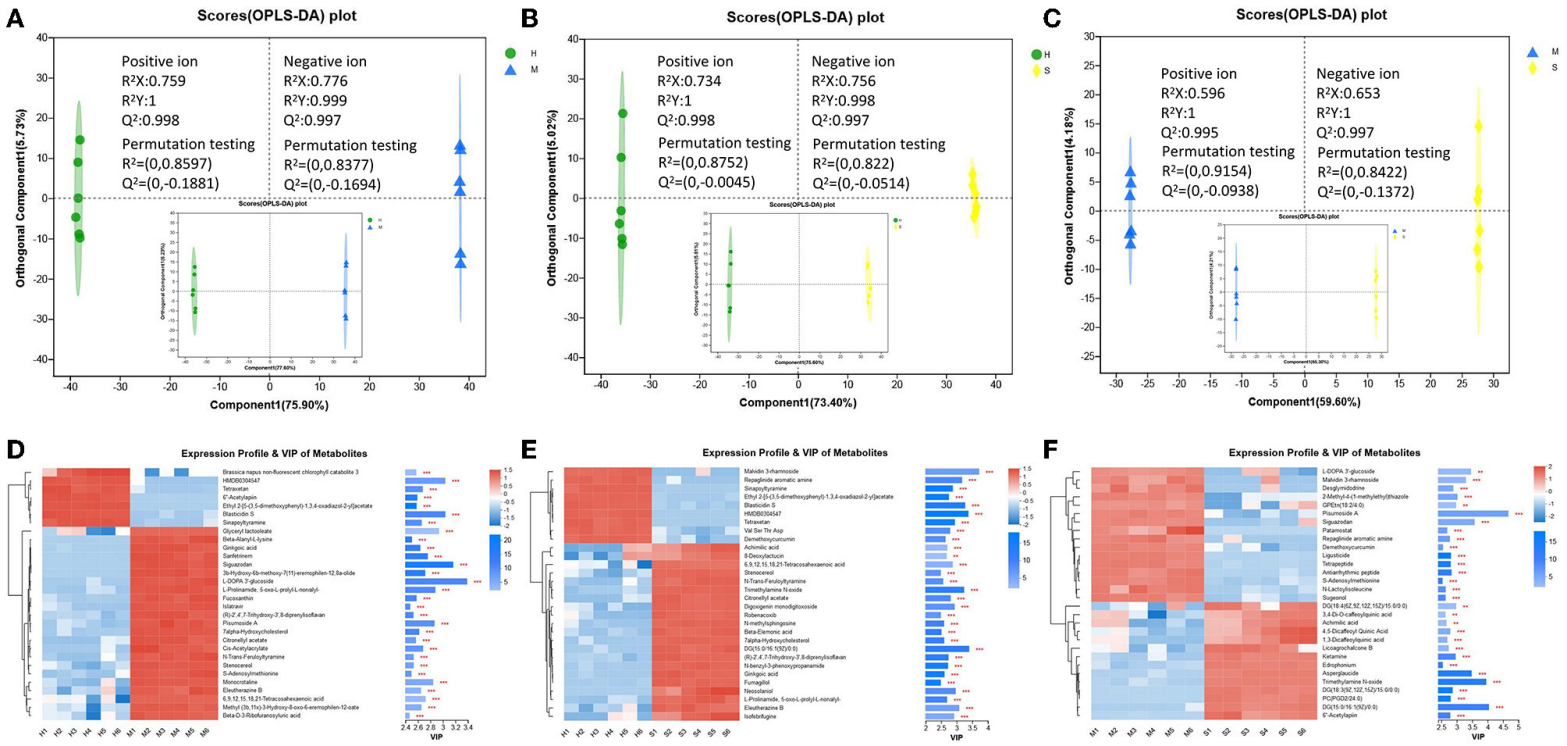
The OPLS-DA plot and expression profile and VIP of metabolites between different comparison groups. **(A–C)** The OPLS-DA plot of different comparison groups (the larger plot indicated the positive ion modes, and the smaller plot indicated the negative ion modes). **(D–F)** The expression profile and VIP of metabolites of different comparison groups.

In this study, the three groups could be better distinguished by OPLS-DA analysis in both positive and negative modes, and the OPLS-DA model was feasible, and the metabolites also differed significantly between the three comparison groups (Figures 3A–C). All the identified metabolites were used for screening differential metabolites according to the VIP > 1 and *p* < 0.05, and the results of the three comparison groups are presented in Figures 3D–F. A total of 1,594 differential metabolites were identified among the H, M, and S treatments (Supplementary Figure 4). Of these, a total of 1,050, 1,036, and 956 differential metabolites were identified in H vs. M, H vs. S, and S vs. M comparisons, respectively (Supplementary Figures 5A–C). Based on these differential metabolites, the analysis of KEGG compounds classification showed that a total of 98 differential metabolites were detected among the three treatments (Table 1). Among these, there were 62, 53, and 69 differential metabolites in H vs. M, H vs. S, and S vs. M comparisons, respectively, which mainly included phospholipids, carboxylic acids, monosaccharides, amino acids, and vitamins (Supplementary Figures 5D, E). Furthermore, KEGG enrichment analysis revealed that a total of 85, 79, and 46 differential metabolites in H vs. M, H vs. S, and S vs. M comparisons were significantly enriched in different pathways, respectively (*p* < 0.05; Supplementary Figure 6; Supplementary Table 3). Notably, a total of 28, 51, and 19 differential metabolites presented the highest abundance in H, M, and S treatments, respectively (Table 1).

**Table 1:** Details of 98 differential metabolites identified by LC-MS among the H, M, and S treatments.

**Metabolite**	**Formula**	**H_vs_M**			**H_vs_S**			**S_vs_M**			**H**	**M**	**S**
		**VIP_PLS-DA**	**FC(H/M)**	***P*** **-value**	**VIP_PLS-DA**	**FC(H/S)**	***P*** **-value**	**VIP_PLS-DA**	**FC(S/M)**	***P*** **-value**			
12-Keto-tetrahydro-leukotriene B4	C_20_H_34_O_4_	1.2881	0.9195	2.087E-14	0.6172	0.9814	2.679E-07	1.4599	0.9369	2.989E-14	6.44 ± 0.01	**7** **±0**	6.56 ± 0.01
2-Lysophosphatidylcholine (22)	C_26_H_54_NO_7_P	0.5838	0.9834	1.691E-07	0.5809	1.015	0.00000211	1.1207	0.9688	1.977E-10	7.22 ± 0.01	**7.34** **±0**	7.12 ± 0.01
2-Methoxyestradiol (23)	C_19_H_26_O_3_	1.3934	0.8841	2.783E-09	0.7878	0.9609	0.0000174	1.4808	0.9201	2.552E-09	5.09 ± 0.03	**5.75** **±0.02**	5.29 ± 0.01
3-Methyl-2-oxovaleric acid (24)	C_6_H_10_O_3_	0.6357	1.024	0.000008318	1.3568	1.1	6.261E-13	1.4226	0.9309	9.081E-10	**6.27** **±0.01**	6.13 ± 0.02	5.7 ± 0.01
5,6-Dihydroxyprostaglandin F1a	C_20_H_36_O_7_	1.0454	0.9255	2.635E-07	1.4826	0.8747	1.044E-11	1.1795	1.0581	4.786E-07	4.76 ± 0.02	5.14 ± 0.03	**5.44** **±0.01**
Adenosine (25)	C_10_H_13_N_5_O_4_	1.5127	1.134	2.726E-13	1.3645	1.0918	2.147E-12	1.107	1.0387	2.882E-07	**6.71** **±0.01**	5.92 ± 0.01	6.14 ± 0.01
Aspartic acid (26)	C_4_H_7_NO_4_	1.0809	1.0817	5.692E-11	0.8941	1.0503	7.28E-08	0.808	1.0299	0.00001277	**5.28** **±0.01**	4.88 ± 0.01	5.03 ± 0.02
Beta-alanine (27)	C_3_H_7_NO_2_	1.003	0.9474	9.56E-09	0.7627	0.9704	0.000003878	0.8361	0.9763	0.00001803	6.23 ± 0.01	**6.57** **±0.01**	6.42 ± 0.02
Brassinolide (28)	C_28_H_48_O_6_	1.135	0.9405	1.08E-10	0.5866	0.9825	0.0003741	1.217	0.9573	0.000000167	6.95 ± 0.01	**7.39** **±0.01**	7.08 ± 0.02
Bufalin (29)	C_24_H_34_O_4_	1.5954	0.8489	8.506E-09	0.6417	0.9666	0.009036	1.9781	0.8783	2.015E-08	5.03 ± 0.04	**5.92** **±0.03**	5.2 ± 0.03
Butyric acid (30)	C_4_H_8_O_2_	1.4546	0.8526	1.137E-10	1.4403	0.8711	1.08E-10	0.6796	0.9788	0.002375	4.26 ± 0.02	**4.99** **±0.02**	4.89 ± 0.02
Cephamycin C	C_16_H_22_N_4_O_9_S	0.6106	1.0277	0.01001	1.5627	1.1289	9.214E-07	1.7268	0.9104	0.00002892	**6.74** **±0.03**	6.56 ± 0.05	5.97 ± 0.07
Cyclic AMP (31)	C_10_H_12_N_5_O_6_P	1.2389	0.9085	2.538E-11	1.3459	0.9025	1.746E-12	0.3727	1.0066	0.004309	5.2 ± 0.01	5.72 ± 0.01	**5.76** **±0**
Cytidine (32)	C_9_H_13_N_3_O_5_	1.4123	0.89	1.256E-14	0.9494	0.9532	6.315E-11	1.5172	0.9337	3.443E-13	5.58 ± 0.01	**6.27** **±0.01**	5.86 ± 0.01
Cytosine	C_4_H_5_N_3_O	1.4064	0.8878	1.62E-14	0.9314	0.9537	1.881E-11	1.526	0.9309	4.702E-13	5.42 ± 0.01	**6.1** **±0.01**	5.68 ± 0
D-(+)-Trehalose (33)	C_12_H_22_O_11_	0.5726	1.0144	0.000004033	1.181	1.0552	3.261E-10	1.2278	0.9613	5.058E-09	**8.31** **±0.01**	8.19 ± 0.01	7.88 ± 0.02
Dehydroepiandrosterone Sulfate (34)	C_19_H_28_O_5_S	0.8396	0.9578	9.171E-08	1.1875	1.0849	1.665E-11	1.8122	0.8829	1.306E-11	5.59 ± 0.01	**5.83** **±0.02**	5.15 ± 0.01
Deoxyuridine (35)	C_9_H_12_N_2_O_5_	2.0087	0.7362	5.484E-09	2.1873	0.7297	2.561E-09	0.3052	1.0089	0.2558	3.95 ± 0.07	5.36 ± 0.03	**5.41** **±0.02**
Diosgenin (36)	C_27_H_42_O_3_	1.9033	0.8049	1.197E-12	1.9001	0.8255	4.764E-12	0.9343	0.975	7.904E-08	5.18 ± 0.03	**6.43** **±0.01**	6.27 ± 0.01
Equilenin	C_18_H_18_O_2_	1.1279	0.9025	0.00001071	1.0862	1.1109	0.001193	2.1883	0.8124	0.000001781	4.37 ± 0.05	**4.84** **±0.03**	3.93 ± 0.09
Ergocalciferol (37)	C_28_H_44_O	2.3201	0.7179	5.527E-12	2.4369	0.7253	8.566E-12	0.5711	0.9897	0.0002349	4.75 ± 0.05	**6.62** **±0.01**	6.55 ± 0.01
Estrone (38)	C_18_H_22_O_2_	1.4975	0.8296	1.423E-07	1.7527	0.7979	1.351E-08	0.8834	1.0398	0.0001423	3.81 ± 0.06	4.6 ± 0.02	**4.78** **±0.02**
Ganosporeric acid A	C_30_H_38_O_8_	0.8042	1.0653	0.02369	1.2672	1.1044	0.00004382	0.6269	0.9645	0.2246	**5.65** **±0.03**	5.3 ± 0.13	5.11 ± 0.07
Geldanamycin (39)	C_29_H_40_N_2_O_9_	1.2966	1.1057	1.713E-07	1.1005	1.0669	1.804E-07	0.868	1.0364	0.003196	**6.14** **±0.01**	5.55 ± 0.04	5.75 ± 0.03
Gluconic acid (40)	C_6_H_12_O_7_	0.8804	0.9614	2.142E-07	1.0331	0.9525	1.938E-08	0.454	1.0093	0.02008	6.74 ± 0.01	7.01 ± 0.02	**7.08** **±0.02**
GPCho (22:5/14:1)	C_44_H_76_NO_8_P	0.1699	0.996	0.2083	1.13	1.0669	0.000000014	1.5156	0.9336	2.189E-10	6.27 ± 0.02	**6.3** **±0.01**	5.88 ± 0.02
Guanine (41)	C_5_H_5_N_5_O	1.0697	0.9382	1.512E-12	0.3387	0.9929	0.001421	1.3975	0.9449	5.398E-12	6.03 ± 0.01	**6.43** **±0.01**	6.08 ± 0.01
Guanosine (42)	C_10_H_13_N_5_O_5_	1.127	0.9375	2.015E-13	0.4325	0.9907	0.000006202	1.3355	0.9463	3.445E-13	6.46 ± 0.01	**6.9** **±0.01**	6.53 ± 0
Ketoleucine (43)	C_6_H_10_O_3_	0.3546	1.0098	0.003071	0.9446	1.0514	1.795E-10	1.0261	0.9604	2.946E-08	**5.69** **±0.01**	5.63 ± 0.01	5.41 ± 0.01
L-(+)-Arginine (44)	C_6_H_14_N_4_O_2_	1.5255	1.1735	1.83E-11	1.3891	1.1199	1.621E-11	1.0703	1.0479	0.00001412	**5.47** **±0.01**	4.66 ± 0.02	4.88 ± 0.02
L-Alanine (45)	C_3_H_7_NO_2_	0.3492	1.0076	0.00005126	0.946	1.0466	4.718E-11	1.113	0.9627	9.76E-11	**6.09** **±0.01**	6.04 ± 0	5.81 ± 0.01
L-Dopa (46)	C_9_H_11_NO_4_	1.2629	0.8902	1.162E-07	1.3286	0.8906	9.824E-09	0.0106	0.9996	0.9694	4.51 ± 0.02	**5.07** **±0.03**	5.06 ± 0.02
Leukotriene B4 (47)	C_20_H_32_O_4_	0.2656	0.9877	0.2412	0.9371	0.9432	0.0001077	1.0264	1.0471	0.0000528	5.05 ± 0.04	5.11 ± 0.03	**5.35** **±0.02**
L-Glutamine (48)	C_5_H_10_N_2_O_3_	1.6822	1.1632	4.519E-17	1.933	1.1931	5.136E-19	0.9045	0.975	2.623E-09	**6.97** **±0**	5.99 ± 0.01	5.84 ± 0
L-Phenylalanine (49)	C_9_H_11_NO_2_	1.7595	1.1779	4.929E-16	1.7053	1.1415	3.182E-15	1.02	1.0319	0.000000044	**7.09** **±0.01**	6.02 ± 0.01	6.21 ± 0.01
L-Proline (50)	C_5_H_9_NO_2_	0.8701	1.0392	2.109E-10	1.0631	1.0516	2.859E-10	0.6259	0.9881	0.0001529	**7.01** **±0.01**	6.74 ± 0.01	6.66 ± 0.01
L-Threonine (51)	C_4_H_9_NO_3_	0.9301	1.0537	5.07E-13	1.1132	1.0683	3.241E-12	0.6213	0.9863	0.0000209	**5.87** **±0**	5.57 ± 0	5.49 ± 0.01
L-Tryptophan (52)	C_11_H_12_N_2_O_2_	1.1645	1.0863	5.313E-13	1.1532	1.0767	7.973E-11	0.4141	1.009	0.005414	**5.8** **±0.01**	5.34 ± 0.01	5.39 ± 0.01
L-Tyrosine (53)	C9H11NO3	1.3773	1.126	1.63E-12	1.4919	1.1366	3.685E-10	0.32	0.9906	0.1615	**5.77** **±0.01**	5.12 ± 0.01	5.07 ± 0.03
LysoPC (15:0/0:0)	C_23_H_48_NO_7_P	0.5986	0.9786	3.555E-07	0.6493	1.0227	5.261E-08	1.1922	0.9569	1.796E-10	5.86 ± 0.01	**5.99** **±0.01**	5.73 ± 0.01
LysoPC (16:0/0:0)	C_24_H_50_NO_7_P	0.4884	0.9893	3.269E-07	0.5682	1.0129	3.924E-08	1.0086	0.9767	1.431E-10	7.85 ± 0	**7.93** **±0.01**	7.75 ± 0
LysoPC [18:3 (6Z, 9Z, 12Z)/0:0]	C_26_H_48_NO_7_P	0.6051	1.017	0.000002267	1.1171	1.0499	7.677E-10	1.1627	0.9686	2.677E-09	**8.01** **±0.01**	7.88 ± 0.01	7.63 ± 0.01
Malonic acid (54)	C_3_H_4_O_4_	1.5618	1.171	8.85E-11	1.1371	1.0809	0.000007453	1.3505	1.0833	0.00001208	**5.7** **±0.02**	4.87 ± 0.02	5.28 ± 0.05
Mitomycin (55)	C_15_H_18_N_4_O_5_	0.2909	0.9915	0.03008	0.8314	1.0439	1.828E-07	1.1179	0.9498	1.123E-07	5.23 ± 0.01	**5.28** **±0.01**	5.01 ± 0.01
Muramic acid	C_9_H_17_NO_7_	0.9483	0.9526	4.024E-10	0.373	1.0094	0.009346	1.4316	0.9437	9.616E-11	6.31 ± 0.01	**6.63** **±0**	6.26 ± 0.01
N-Acetylmannosamine (56)	C_8_H_15_NO_6_	0.7634	0.9631	5.354E-09	1.094	0.9365	5.816E-11	0.9232	1.0284	2.493E-07	5.36 ± 0.01	5.56 ± 0.01	**5.72** **±0.01**
N-Acetylmuramate	C_11_H_19_NO_8_	1.296	0.8928	2.984E-09	0.4641	0.9802	0.02189	1.6295	0.9109	1.657E-08	4.9 ± 0.03	**5.49** **±0.02**	5 ± 0.03
N-Acetylneuraminic acid (57)	C_11_H_19_NO_9_	0.7658	1.0304	9.489E-09	1.1511	1.0643	1.929E-09	0.9844	0.9682	0.000003255	**6.87** **±0**	6.67 ± 0.01	6.46 ± 0.02
Nalidixic acid (58)	C_12_H_12_N_2_O_3_	1.1579	0.9087	2.029E-07	0.95	1.0694	0.0000251	1.9229	0.8497	2.164E-10	4.67 ± 0.03	**5.14** **±0.02**	4.37 ± 0.02
Neomycin sulfate (59)	C_23_H_46_N_6_O_13_	1.1703	0.9216	0.00001831	0.2307	0.9901	0.4427	1.56	0.9308	1.591E-07	6.02 ± 0.07	**6.53** **±0.01**	6.08 ± 0.03
Norepinephrine (60)	C_8_H_11_NO_3_	1.5133	0.8338	5.22E-09	1.2365	0.8893	7.604E-07	1.1774	0.9376	2.197E-07	3.95 ± 0.04	**4.74** **±0.01**	4.44 ± 0.02
Ouabain (61)	C_29_H_44_O_12_	1.4754	0.8844	3.256E-11	1.399	0.9031	1.709E-10	0.7647	0.9793	0.00004191	5.68 ± 0.02	**6.42** **±0.01**	6.29 ± 0.01
Oxoglutaric acid	C_5_H_6_O_5_	1.4058	1.1129	1.327E-09	1.9174	1.2056	1.318E-11	1.4795	0.9231	2.862E-08	**6.69** **±0.03**	6.01 ± 0.02	5.54 ± 0.02
PA [14:1 (9Z)/20:4 (8Z, 11Z, 14Z, 17Z)]	C_37_H_63_O_8_P	0.9727	1.0565	1.243E-10	1.1752	1.0733	6.158E-10	0.6503	0.9844	0.0007635	**6.17** **±0.01**	5.84 ± 0.01	5.75 ± 0.02
PA [16:1 (9Z)/22:4 (7Z, 10Z, 13Z, 16Z)]	C_41_H_71_O_8_P	0.8219	0.9587	0.0001234	1.1136	0.9381	2.151E-07	0.7006	1.0219	0.005787	5.99 ± 0.03	6.25 ± 0.03	**6.38** **±0.02**
PA [18:3 (9Z, 12Z, 15Z)/18:3 (6Z, 9Z, 12Z)]	C_39_H_65_O_8_P	1.2009	1.0922	0.000003993	0.943	1.0462	1.629E-07	1.0678	1.044	0.001126	**6.25** **±0.02**	5.73 ± 0.06	5.98 ± 0.01
PA [18:3 (9Z, 12Z, 15Z)/22:1 (13Z)]	C_43_H_77_O_8_P	1.2323	0.9288	1.037E-09	0.7468	0.9736	0.00007617	1.3683	0.954	4.377E-09	6.93 ± 0.02	**7.46** **±0.01**	7.12 ± 0.02
PA [18:4 (6Z, 9Z, 12Z, 15Z)/18:3 (6Z, 9Z, 12Z)]	C_39_H_63_O_8_P	1.2772	1.0951	1.704E-09	1.1746	1.0687	2.409E-09	0.8253	1.0247	0.0009023	**6.57** **±0.01**	6 ± 0.03	6.14 ± 0.02
PA [20:0/20:4 (8Z, 11Z, 14Z, 17Z)]	C_43_H_77_O_8_P	1.4539	0.8724	2.996E-08	1.7141	0.8499	7.768E-09	0.8744	1.0265	0.0001046	5.1 ± 0.05	5.85 ± 0.01	**6** **±0.02**
PA [20:1 (11Z)/22:5 (7Z, 10Z, 13Z, 16Z, 19Z)]	C_45_H_77_O_8_P	0.9406	0.954	4.54E-08	0.2938	1.0081	0.09852	1.4076	0.9464	2.542E-08	6.5 ± 0.02	**6.81** **±0.01**	6.44 ± 0.02
PA [20:3 (8Z, 11Z, 14Z)/22:2 (13Z, 16Z)]	C_45_H_79_O_8_P	1.0355	0.9398	3.986E-09	0.5818	1.0228	0.001128	1.5376	0.9188	3.757E-09	5.74 ± 0.02	**6.11** **±0.01**	5.62 ± 0.02
PA [20:4 (8Z, 11Z, 14Z, 17Z)/22:2 (13Z, 16Z)]	C_45_H_77_O_8_P	1.0387	0.9434	3.526E-10	0.5468	1.0172	0.0001249	1.5058	0.9274	2.112E-10	6.14 ± 0.01	**6.51** **±0.01**	6.04 ± 0.01
PA [22:1 (13Z)/18:3 (9Z, 12Z, 15Z)]	C_43_H_77_O_8_P	1.3305	0.9088	2.803E-10	0.7893	0.9672	0.000005046	1.3792	0.9397	1.305E-08	6.04 ± 0.02	**6.65** **±0.02**	6.25 ± 0.02
PA [22:6 (4Z, 7Z, 10Z, 13Z, 16Z, 19Z)/20:1 (11Z)]	C_45_H_75_O_8_P	0.654	0.981	3.528E-07	0.7743	1.024	3.39E-08	1.3605	0.958	1.501E-09	7.9 ± 0.01	**8.05** **±0.01**	7.71 ± 0.01
PA (8:0/12:0)	C_23_H_45_O_8_P	1.3973	0.8562	5.291E-08	0.4949	0.9821	1.267E-09	1.8206	0.8718	0.000000155	4.11 ± 0	**4.81** **±0.05**	4.19 ± 0
PC [14:0/22:5 (7Z, 10Z, 13Z, 16Z, 19Z)]	C_44_H_78_NO_8_P	0.5197	0.9815	0.01155	0.7204	1.0269	0.0006396	1.2483	0.9558	0.0001697	7.11 ± 0.01	**7.24** **±0.04**	6.92 ± 0.04
PC [14:0/22:6 (4Z, 7Z, 10Z, 13Z, 16Z, 19Z)]	C_44_H_76_NO_8_P	0.5838	0.9812	0.00004699	0.8575	1.0349	2.131E-07	1.3986	0.9481	8.681E-09	6.79 ± 0.01	**6.92** **±0.02**	6.56 ± 0.01
PC [16:0/18:3 (9Z, 12Z, 15Z)]	C_42_H_78_NO_8_P	0.688	0.9739	0.0004022	0.4924	1.0123	0.0006259	1.1911	0.9621	0.00002371	7.16 ± 0.01	**7.35** **±0.04**	7.07 ± 0.01
PC [20:2 (11Z, 14Z)/14:0]	C_42_H_80_NO_8_P	1.572	0.8191	0.0008414	0.9179	0.9048	0.08466	1.3406	0.9054	0.03823	4.67 ± 0.18	**5.69** **±0.13**	5.16 ± 0.19
PE [22:4 (7Z, 10Z, 13Z, 16Z)/22:6 (4Z, 7Z, 10Z, 13Z, 16Z, 19Z)]	C_49_H_78_NO_8_P	0.1155	1.0034	0.4959	0.893	0.9575	0.0001779	1.197	1.0479	0.0001871	6.18 ± 0.02	6.16 ± 0.03	**6.45** **±0.04**
PG (16:0/16:0)	C_38_H_75_O_10_P	0.7731	1.0509	0.005275	0.5675	0.967	0.08432	1.3345	1.0867	0.001889	5.6 ± 0.04	5.33 ± 0.06	**5.79** **±0.09**
PG [16:1 (9Z)/18:0]	C_40_H_77_O_10_P	1.8157	0.812	2.696E-10	1.2541	0.9084	0.000002623	1.8825	0.8939	2.97E-09	4.96 ± 0.04	**6.11** **±0.02**	5.46 ± 0.03
PG [18:2 (9Z, 12Z)/16:0]	C_40_H_75_O_10_P	1.4936	0.8873	9.34E-13	0.9864	0.9532	1.386E-08	1.6176	0.9309	3.772E-12	6.09 ± 0.02	**6.86** **±0.01**	6.39 ± 0.01
PG [22:6 (4Z, 7Z, 10Z, 13Z, 16Z, 19Z)/20:3 (8Z, 11Z, 14Z)]	C_48_H_77_O_10_P	1.2207	0.9237	3.143E-09	1.2945	0.9223	7.805E-10	0.0754	1.0015	0.7435	6.2 ± 0.01	6.71 ± 0.02	**6.72** **±0.02**
PGF2alpha (62)	C_20_H_34_O_5_	1.3698	0.9012	8.128E-15	0.8094	0.9659	1.035E-08	1.4399	0.933	1.408E-11	5.8 ± 0.01	**6.43** **±0.01**	6 ± 0.01
Phenethylamine (63)	C_8_H_11_N	1.5871	0.8151	3.489E-11	1.0509	0.9168	0.000001844	1.6969	0.8891	8.698E-10	3.86 ± 0.03	**4.73** **±0.01**	4.21 ± 0.02
Pimelic acid	C_7_H_12_O_4_	0.7622	1.0428	0.0001089	1.854	1.2503	8.601E-09	2.0098	0.834	8.096E-08	**5.39** **±0.03**	5.17 ± 0.03	4.31 ± 0.06
Pregnenolone (64)	C_21_H_32_O_2_	1.0552	1.0677	6.302E-10	0.1636	1.0029	0.1285	1.4284	1.0646	1.846E-10	**6.13** **±0.01**	5.74 ± 0.01	6.11 ± 0
Prostaglandin A1 (65)	C_20_H_32_O_4_	1.2637	0.9188	1.22E-11	0.7696	0.9715	7.029E-08	1.4282	0.9457	2.111E-10	6.28 ± 0.01	**6.84** **±0.01**	6.47 ± 0.01
Prostaglandin B1	C_20_H_32_O_4_	1.1885	0.9278	1.268E-13	0.7168	0.9742	7.559E-08	1.2353	0.9523	7.229E-12	6.16 ± 0.01	**6.64** **±0**	6.33 ± 0.01
Prostaglandin B2	C_20_H_30_O_4_	1.2817	0.8996	8.161E-11	0.9739	0.9438	2.87E-08	1.1156	0.9531	5.584E-09	5.03 ± 0.02	**5.59** **±0.01**	5.33 ± 0.01
Prostaglandin D2 (66)	C_20_H_32_O_5_	0.4911	0.9827	0.001075	1.2123	0.9285	1.142E-10	1.3668	1.0584	4.715E-08	5.81 ± 0.01	5.91 ± 0.02	**6.26** **±0.01**
Prostaglandin E1 (67)	C_20_H_34_O_5_	1.4184	0.8865	1.212E-10	1.7529	0.8545	8.69E-12	1.119	1.0375	3.573E-08	5.47 ± 0.02	6.17 ± 0.01	**6.4** **±0.01**
Prostaglandin F1a	C_20_H_36_O_5_	1.8087	0.782	0.00000566	2.3332	0.718	2.109E-07	1.6338	1.0891	3.552E-08	4.32 ± 0.14	5.52 ± 0.03	**6.01** **±0.02**
Prostavasin (68)	C_20_H_34_O_5_	0.5111	0.9795	0.005108	1.2199	0.9264	3.93E-08	1.3557	1.0574	3.157E-08	5.79 ± 0.03	5.91 ± 0.02	**6.25** **±0.01**
PS [18:1 (9Z)/20:1 (11Z)]	C_44_H_82_NO_10_P	1.9575	0.7833	1.166E-07	1.5521	0.8621	0.00001838	1.7747	0.9085	8.99E-10	4.92 ± 0.1	**6.28** **±0.02**	5.71 ± 0.02
Pyridoxine (69)	C_8_H_11_NO_3_	0.5143	1.0187	0.0006751	0.5471	0.9812	0.002176	1.0699	1.0383	0.00001543	5.93 ± 0.02	5.82 ± 0.02	**6.05** **±0.02**
Riboflavin (70)	C_17_H_20_N_4_O_6_	0.9617	0.9443	3.59E-10	0.5364	0.9834	0.000004509	1.1174	0.9602	3.432E-08	5.46 ± 0.01	**5.78** **±0.01**	5.55 ± 0.01
S-adenosyl-L-methioninamine	C_14_H_24_N_6_O_3_S+2	2.0755	0.7605	1.751E-11	2.1985	0.7568	2.559E-12	0.1811	1.0049	0.4756	4.66 ± 0.03	6.13 ± 0.03	**6.16** **±0.02**
S-Adenosylmethionine (71)	C_15_H_23_N_6_O_5_S+	2.4903	0.6324	2.639E-09	1.5273	0.822	0.00004823	2.531	0.7693	2.298E-10	3.66 ± 0.11	**5.79** **±0.02**	4.45 ± 0.05
Serotonin (72)	C_10_H_12_N_2_O	1.4567	1.1507	5.291E-13	1.3166	1.1032	1.48E-12	1.0708	1.0431	8.973E-09	**5.61** **±0.01**	4.88 ± 0.01	5.09 ± 0.01
Suberic acid	C_8_H_14_O_4_	1.0863	0.9267	1.068E-09	1.2671	0.9111	3.023E-11	0.6243	1.0171	0.0003453	5.1 ± 0.01	5.51 ± 0.01	**5.6** **±0.01**
Sucrose (73)	C_12_H_22_O_11_	0.8143	1.0305	0.000002013	1.1728	1.0573	9.601E-09	0.9264	0.9747	0.00004525	**7.97** **±0.01**	7.74 ± 0.02	7.54 ± 0.02
Thromboxane B2	C_20_H_34_O_6_	1.1626	0.9171	2.354E-09	1.0983	0.9321	3.497E-12	0.5755	0.9839	0.004316	5.14 ± 0	**5.6** **±0.02**	5.51 ± 0.01
Thyrotropin-releasing factor (74)	C_16_H_22_N_6_O_4_	1.5535	0.8584	5.68E-10	1.1585	0.9216	1.523E-07	1.3764	0.9314	1.753E-08	5.01 ± 0.03	**5.83** **±0.02**	5.43 ± 0.01
Trehalose (75)	C_12_H_22_O_11_	0.5691	1.0165	0.00005649	1.0235	1.0443	5.364E-08	1.016	0.9734	0.00001035	**7.64** **±0.01**	7.51 ± 0.01	7.31 ± 0.02
Uracil (76)	C_4_H_4_N_2_O_2_	1.0516	0.9392	8.617E-13	0.6838	0.9761	4.514E-08	1.1451	0.9622	4.122E-10	5.92 ± 0.01	**6.3** **±0.01**	6.07 ± 0.01
Uridine (77)	C_9_H_12_N_2_O_6_	1.0592	0.9482	1.785E-10	0.4745	0.9895	0.000007563	1.2138	0.9582	3.083E-09	7 ± 0	**7.39** **±0.01**	7.08 ± 0.01

The bold values indicated the highest abundance.

#### 3.3.1. Saccharides

**Figure 4:**
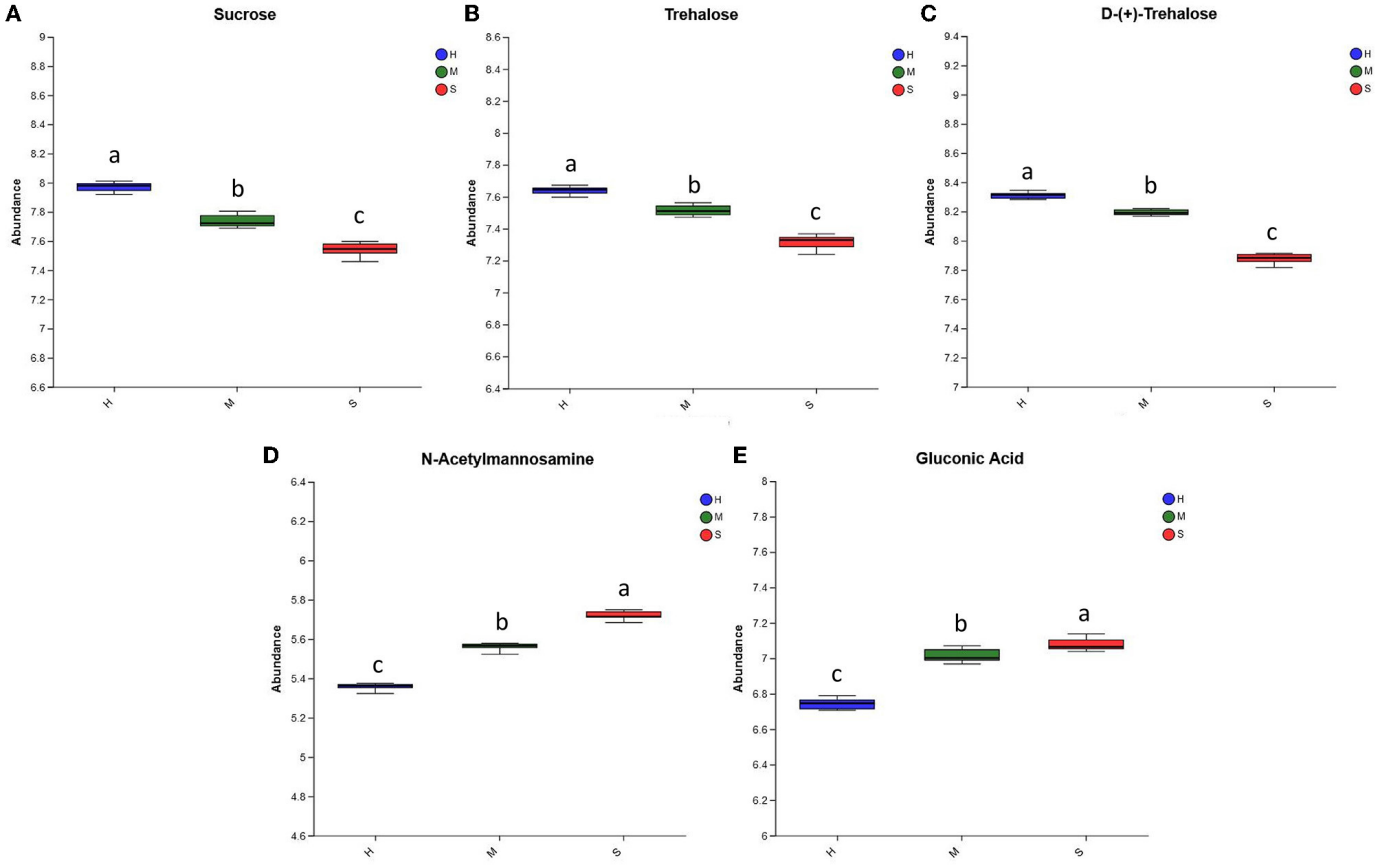
The relative abundance of the differential saccharides among the H, M, and S treatments. **(A–E)** The abundances of Sucrose, Trehalose, D-(+)-Trehalose, NAcetylmannosamine, and Gluconic Acid among the three treatments, respectively. The treatments containing the same letters indicated no significant differences (*p* > 0.05), while the treatments containing different letters indicated significant differences (*p* < 0.05).

As shown in Figures 4A–C, the abundances of sucrose, trehalose, and D-(+)-trehalose were ranked as H>M>S, suggesting that the H and M treatments favored the formation and accumulation of these three substances in comparison with the S treatment. Meanwhile, the abundances of gluconic acid and N-acetylmannosamine under the three treatments were ranked as S>M>H, indicating that the S and M treatments were more conducive to the formation and accumulation of these two substances in comparison to the H treatment (Figures 4D, E).

#### 3.3.2. Amino acids

**Figure 5:**
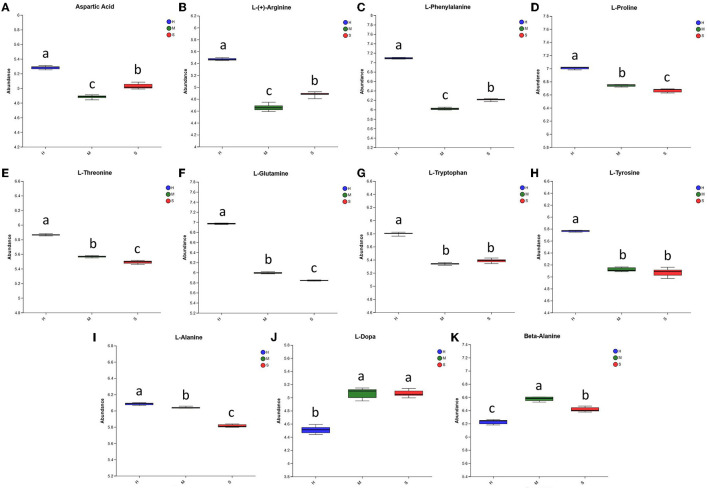
The relative abundance of the differential amino acids among the H, M, and S treatments. **(A–K)** The abundances of Aspartic Acid, L-(+)-Arginine, L-Phenylalanine, LProline, L-Threonine, L-Glutamine, L-Tryptophan, L-Tyrosine, L-Alanine, L-Dopa, and Beta-Alanine among the three treatments, respectively. The treatments containing the same letters indicated no significant differences (*p* > 0.05), while the treatments containing different letters indicated significant differences (*p* < 0.05).

Amino acids are important nutrients for *P. palustre*. Here, we identified 11 differential amino acids, including L-dopa, L-proline, L-alanine, L-glutamine, L-phenylalanine, L-threonine, L-(+)-arginine, beta-alanine, L-tryptophan, L-tyrosine, and aspartic Acid. As shown in Figure 5, the relative abundances of L-dopa and L-alanine in the H treatment were significantly lower than those of the M and S treatments, whereas the relative abundances of the other nine amino acids in the H treatment were dramatically higher than those of the M and S treatments. This suggested that the H treatment could promote the amino acid content of *P. palustre*. In particular, aspartic acid and glutamic acid are delicious amino acids, and alanine, serine, and glycine are sweet amino acids in *P. palustre* (22, 78). In our investigation, the abundances of aspartic acid and L-alanine in the H treatment were significantly increased compared with the M and S treatments, while the beta-alanine presented the lowest abundance in the H treatment.

### 3.4. Volatile compounds analysis based on HS-GC-MS

In this study, we also used the HS-GC-MS technique to investigate the effects of different processing treatments on the volatile components of *P. palustre*. The results showed that a total of 242 metabolites were identified by HS-GC-MS. The analysis of correlation, PCA, Venn, and PLS-DA indicated that the data were reliable and available (Supplementary Figure 7). Based on these metabolites, in total, 55 volatile substances were identified in HMDB, including organic oxygen compounds (17, 30.91%), lipids and lipid-like molecules (15, 27.27%), organic acids and derivatives (8, 14.55%), organoheterocyclic compounds (8, 14.55%), benzenoids (6, 10.91%), and hydrocarbons (1, 1.82%; Supplementary Figure 8A; Supplementary Table 4). Among these, carbonyl compounds, fatty acids and conjugates, and pyrazines accounted for almost half of the volatile substances (27/55; Supplementary Figure 8B).

### 3.5. Differential volatile substances among the different treatments

**Figure 6:**
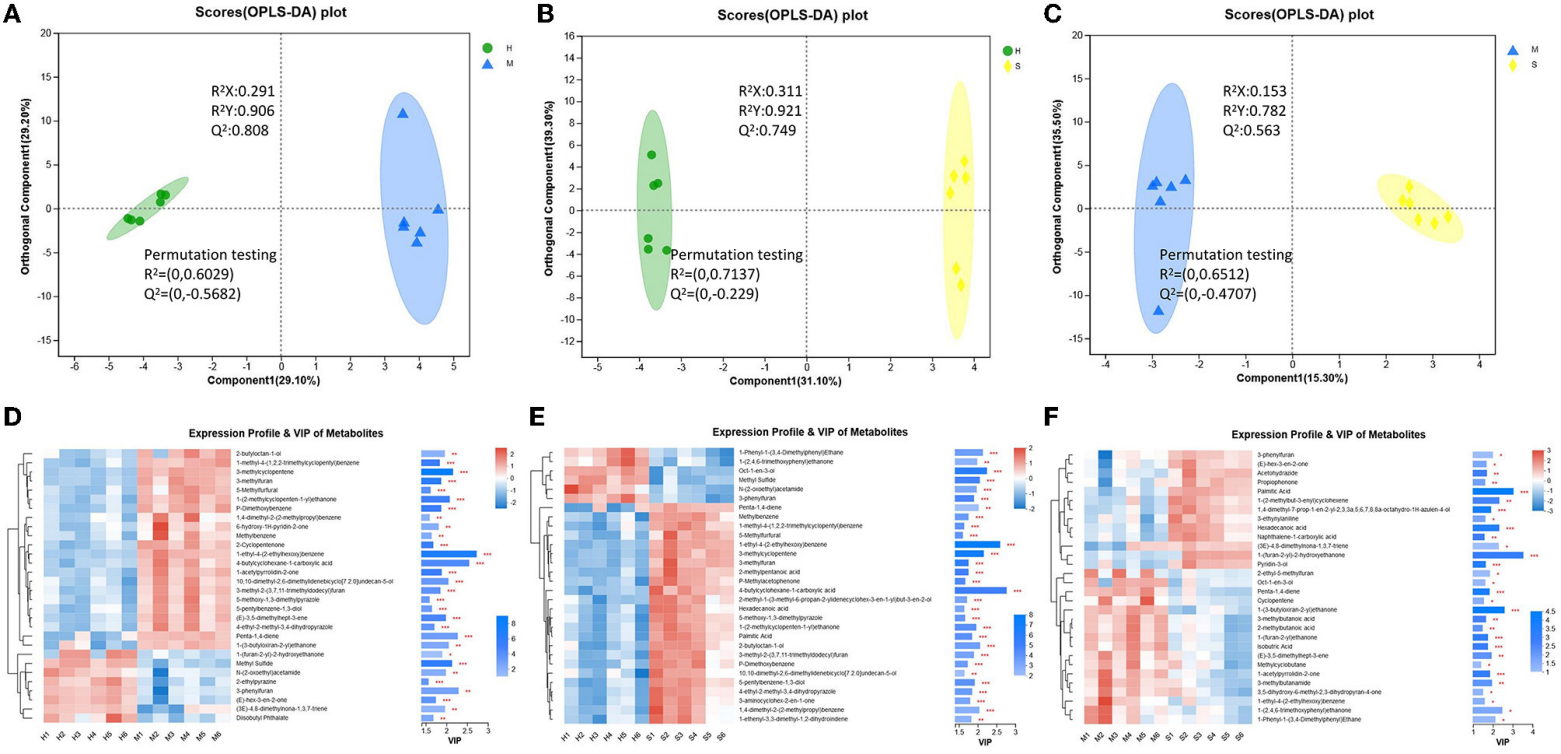
The OPLS-DA plot and expression profile and VIP of volatile substances. **(A–C)** The OPLSDA plot of different comparison groups. **(D–F)** The expression profile and VIP of volatile substances of different comparison groups.

The different groups could be better distinguished by OPLS-DA analysis (Figures 6A–C). All the identified volatile substances were employed for screening differential metabolites according to the VIP > 1 and *p* < 0.05, and the results of the three comparison groups are shown in Figures 6D–F. The results showed that a total of 109 differential volatile substances were detected among the H, M, and S treatments (Supplementary Figure 8C). Of these, there were 70 (23 upregulated and 47 downregulated), 74 (20 upregulated and 54 downregulated), and 44 (22 upregulated and 22 downregulated) differential volatile substances in H vs. M, H vs. S, and S vs. M treatments, respectively (Supplementary Figures 8D–F). Based on these differential volatile substances, we identified 27 differential volatile substances in HMDB (Table 2), including lipids and lipid-like molecules (10, 37.04%), organic oxygen compounds (7, 25.93%), organoheterocyclic compounds (5, 18.52%), benzenoids (2, 7.41%), organic acids and derivatives (2, 7.41%), and hydrocarbons (1, 3.7%) under superclass classification or carbonyl compounds (6, 22.22%), fatty acids and conjugates (6, 22.22%), pyrazines (4, 14.81%), unknown (3, 11.11%), fatty alcohols (2, 7.41%), monoterpenoids (2, 7.41%), benzoic acids and derivatives (1, 3.7%), carboxylic acid derivatives (1, 3.7%), carboxylic acids (1, 3.7%), and olefins (1, 3.7%), under subclass classification (Supplementary Figure 9; Supplementary Table 5). Of these, there were 18, 17, and 11 differential volatile substances in H vs. M, H vs. S, and S vs. M comparisons, respectively. In addition, among the three treatments, there were 11, 9, and 7 differential volatile substances showing the highest abundance in the H, M, and S treatments, respectively (Table 2).

**Table 2:** Details of 27 differential metabolites identified by HS-GC-MS among the H, M, and S treatments.

**Metabolite**	**Quant mass**	**Formula**	**Retention time**	**RSD**	**Score**	**CAS ID**	**H**	**M**	**S**
3-methylcyclopentene	67	C_6_H_10_	1.569	0.070507784	84.7829959	1120-62-3	4.17 ± 0.02	**4.79** **±0.02**	4.72 ± 0.04
2-methylpropanal	72	C_4_H_8_O	1.625	0.068787109	96.96030378	78-84-2	**6.46** **±0.02**	6.27 ± 0.02	6.35 ± 0.03
Acetohydrazide	74	C_2_H_6_N_2_O	1.694	0.0762885	95.19998129	7467-32-5	6.01 ± 0.02	5.86 ± 0.04	**6.09** **±0.03**
Butan-2-one	72.1	C_4_H_8_O	1.933	0.07628602	84.38764977	78-93-3	4.63 ± 0.02	4.67 ± 0.09	**4.87** **±0.05**
2-ethoxyacetic acid	60	C_4_H_8_O_3_	2.049	0.058061107	63.28450641	627-03-2	**3.64** **±0.04**	3.42 ± 0.03	3.56 ± 0.03
2-methylbutanal (79)	57	C_5_H_10_O	2.211	0.074138387	97.24812074	57456-98-1;96-17-3	**6.58** **±0.02**	6.28 ± 0.04	6.33 ± 0.05
2-ethyl-5-methylfuran	95	C_7_H_10_O	3.597	0.130583098	50.40601524	1703-52-2	4.05 ± 0.03	**4.49** **±0.14**	4.16 ± 0.04
Terpinolene (80)	93	C_10_H_16_	4.036	0.082818836	53.3918106	586-62-9	**4.02** **±0.04**	3.75 ± 0.1	3.88 ± 0.06
Hexanal (81)	56	C_6_H_12_O	4.418	0.0853182	92.3798746	66-25-1	4.94 ± 0.03	**5.17** **±0.09**	5.14 ± 0.07
(3E)-4,8-dimethylnona-1,3,7-triene	69	C_11_H_18_	8.618	0.060793879	73.47215285	19945-61-0	**4.91** **±0.03**	4.15 ± 0.24	4.68 ± 0.04
2-Methylpyrazine	94	C_5_H_6_N_2_	8.877	0.057517825	95.00709536	109-08-0	**5.92** **±0.03**	5.59 ± 0.02	5.67 ± 0.02
2,3-Pentanedione	57	C_5_H_8_O_2_	9.369	0.015632225	57.40072131	600-14-6	3.99 ± 0.06	**4.18** **±0.05**	3.99 ± 0.06
2-methylpentanoic acid	98.9	C_6_H_12_O_2_	9.709	0.063366089	83.01372596	22160-39-0;97-61-0	4.27 ± 0.03	4.21 ± 0.49	**4.65** **±0.04**
2,5-dimethylpyrazine (82)	108	C_6_H_8_N_2_	10.203	0.071420374	59.35896515	123-32-0	**5.14** **±0.04**	4.84 ± 0.07	4.82 ± 0.05
2-ethylpyrazine (83)	108	C_6_H_8_N_2_	10.356	0.058623043	92.05534271	13925-00-3	**5.4** **±0.04**	5 ± 0.06	5.08 ± 0.05
Oct-1-en-3-ol	57.2	C_8_H_16_O	13.278	0.076718503	76.40710428	-	**5.44** **±0.02**	5.13 ± 0.1	4.85 ± 0.05
2,6,6-trimethylcyclohexa-1,3-diene-1-carbaldehyde	107.1	C_10_H_14_O	16.439	0.071249392	92.88423312	116-26-7	4.92 ± 0.03	5.12 ± 0.07	**5.12** **±0.04**
3-methylbutanoic acid	60	C_5_H_10_O_2_	17.024	0.062274418	85.17913453	503-74-2	6.02 ± 0.03	**6.09** **±0.03**	5.88 ± 0.04
2-methylbutanoic acid	74	C_5_H_10_O_2_	17.028	0.069051142	81.26700239	116-53-0	5.42 ± 0.03	**5.61** **±0.03**	5.43 ± 0.04
2,6,6-trimethylcyclohex-2-ene-1,4-dione	96.1	C_9_H_12_O_2_	17.081	0.059543144	75.47973568	1125-21-9	4.22 ± 0.04	**4.47** **±0.04**	4.46 ± 0.05
Pyrazine-2-carboxamide	80	C_5_H_5_N_3_O	17.461	0.052748754	59.91665839	98-96-4	4.58 ± 0.03	4.5 ± 0.03	**4.64** **±0.05**
Valeric acid (84)	60.1	C_5_H_10_O_2_	18.642	0.058225562	91.46195141	109-52-4	5.18 ± 0.05	**5.42** **±0.04**	5.31 ± 0.05
2-phenylethanol (85)	91	C_8_H_10_O	19.141	0.055682023	93.71279349	1960/12/8	**5.27** **±0.03**	5.2 ± 0.04	5.09 ± 0.06
2-butyloctan-1-ol	57	C_12_H_26_O	19.318	0.059641269	72.8249512	3913/2/8	3.51 ± 0.08	**4.13** **±0.16**	4.07 ± 0.06
Hexadecanoic acid	74	C_16_H_32_O_2_	21.219	0.058917775	82.23031131	1957/10/3	4.39 ± 0.04	4.33 ± 0.05	**4.76** **±0.06**
Diisobutyl phthalate	148.9	C_16_H_22_O_4_	23.142	0.063856537	89.91536639	84-69-5	**4.61** **±0.1**	4.13 ± 0.06	4.26 ± 0.04
Palmitic acid (86)	73.1	C_16_H_32_O_2_	26.354	0.098671895	87.30590317	1957/10/3	5.28 ± 0.05	5.05 ± 0.08	**5.71** **±0.04**

The bold values indicated the highest abundance.

## 4. Discussion

*P. palustre* mainly contains polysaccharides, flavonoids, triterpenoids, phenolic acid, etc. (11, 87). For evaluating the impact of different processing methods on the quality of *P. palustre*, we determined the contents of total sugar, soluble sugar, total pectin, and total flavonoids. In this study, we adopted three processing methods including sweating (M), tedding (S), and drying (H) to study the influences of different processing methods on the quality of *P. palustre*. The results indicated that the M treatment promoted the accumulation of the contents of total sugar, soluble sugar, and total pectin when compared with the H and S treatments (Figure 2). It was inferred that the M treatment could promote the quality of *P. palustre*. It was also similar to the conclusion of Zhang et al. (16). During the sweating process, the external nutrient supply of *P. palustre* was cut off, and the tissue cells used nutrients such as polysaccharides stored internally for life activities. The polysaccharides might undergo hydrolysis reactions under the catalysis of hydrolytic enzymes, resulting in reduced molecular weight and improved water solubility of polysaccharides, which were manifested by an increase in the water-soluble polysaccharides. However, the total flavonoid content in the M treatment significantly decreased in comparison with the H and S treatments. It was inferred that the M treatment was not favorable to the improvement of total flavonoids. It was consistent with the results of Lu (18). Overall, the M treatment favored the improvement of the polysaccharide quality of *P. palustre*.

The metabolites of each treatment were also analyzed based on LC-MS. The results revealed that a total of 174 metabolites included phospholipids, monosaccharides, fatty acids, carboxylic acids, eicosanoids, amino acids, and vitamins were presented (Supplementary Table 1). Tang et al. (2) identified 184 metabolites in *M. chinensis* Benth by using LC-MS detection, which contained carbohydrates and carbohydrate conjugates, fatty acids and conjugates, eicosanoids, and so on. It was inferred that the number and type of metabolites identified in the present study were similar to the study by Tang et al. (2). This indicated that LC-MS technology was stable and suitable for the identification of *P. palustre* metabolites. Furthermore, we identified 98 differential metabolites among the three treatments. Notably, a total of 28, 51, and 19 metabolites presented the highest abundance in the H, M, and S treatments, respectively (Table 1). We could observe that the M treatment could promote the abundance of more than half of the differential metabolites (51 out of 98) in comparison with the H and S treatments. It was indicated that the M treatment could significantly change the type and abundance of metabolites of *P. palustre*. These changes might have significant impacts on the quality and flavor of this herbal medicine.

The content of polysaccharides is an important index for evaluating the quality of *P. palustre* (11). Zhang et al. (88) found that *P. palustre* polysaccharides consisted of eight monosaccharides, including galacturonic acid, glucose, galactose, xylose, mannose, rhamnose, ribose, and glucuronic acid. In this study, the relative abundances of sucrose, gluconic acid, D-(+)-trehalose, trehalose, and N-acetylmannosamine among the H, M, and S treatments were identified and analyzed. The abundances of gluconic acid and N-acetylmannosamine under three treatments were ranked as S>M>H, and the abundances of D-(+)-trehalose, sucrose, and trehalose were ranked as H>M>S (Figure 4). This suggested that the M treatment was beneficial to the stability of the polysaccharide quality of *P. palustre*. Combined with the results in Figures 2, 4, the M treatment seemed to be more favorable for the stability and improvement of the quality of *P. palustre* polysaccharides, although it decreased the content of total flavonoids.

*P. palustre* is also rich in amino acids, and at least 18 types of amino acids including aspartic acid (Asp), threonine (Thr), serine (Ser), glutamic acid (Glu), glycine (Gly), cysteine (Cys), methionine (Met), valine (Val), isoleucine (Ile), tyrosine (Tyr), phenylalanine (Phe), lysine (Lys), histidine (His), arginine (Arg), proline (Pro), alanine (Ala), leucine (Leu), and tryptophan (Trp) were determined by Liu and Chen (89). Similarly, Su et al. (78) reported 17 types of amino acids in *P. palustre* from different production areas, which were almost the same with the results of Liu and Chen (89). In this study, we identified 11 differential amino acids, such as L-dopa, L-proline, L-alanine, L-glutamine, L-phenylalanine, L-threonine, L-(+)-arginine, beta-alanine, L-tryptophan, L-tyrosine, and aspartic Acid, and the abundances of nine amino acids significantly increased in the H treatment (Figure 5). It was indicated that the H treatments could significantly change the abundances of the most differential amino acids in *P. palustre*. This might be because that the relatively high-temperature condition (50°C) in the H treatment promoted the degradation of protein into amino acids, thereby increasing the expression abundance of amino acids, thus improving their nutritional levels. In addition, since Asp and Glu are delicious amino acids, and Ala, Ser, and Gly are sweet amino acids in *P. palustre* (78). The abundances of aspartic acid and L-alanine in the H treatment were significantly increased compared with the M and S treatments, indicating that the H treatment could promote the flavor level of *P. palustre*. Therefore, in general, the H treatment could improve the nutrition and flavor level of *P. palustre*.

Volatile oil, or essential oil, is a general term used for oily liquids that are volatile and can be distilled with steam (90), and has various effects such as antibacterial (91), anti-inflammatory (92), and antitumor (93). Volatile oil is widely found in herbal medicines such as *P. palustre* (17). Lu et al. (12) identified 24 types of volatile constituents from Taiwan *Mesona chinensis* Benth, while Xu and Wei (94) found 56, 54, and 58 types of volatile flavor compounds in Vietnam, Thailand, and Indonesia, respectively. Wei et al. (95) extracted and detected 59 volatile components from the water-soluble extract of *Mesona Benth* prepared by the solvent extraction method, while Chen et al. (96) identified 40 and 34 volatile components using supercritical CO_2_ extraction and hydrodistillation methods, respectively. Kung et al. (97) determined 56 and 108 volatile components using headspace solid-phase microextraction (HS-SPME) and simultaneous distillation–extraction (SDE), respectively. In this study, a total of 55 volatile substances were identified in HMDB by HS-GC-MS (Supplementary Table 4). It was inferred that different volatile components of *P. palustre* could be obtained from different varieties (or regional sources) and different extraction methods and technologies. Furthermore, the main volatile compounds identified in *P. palustre* were alkenes, alcohols, ketones, aldehydes, and phenols (95) or alkenes, ketones, alkanes, and fatty acids (98), or terpenoids and carbonyl compounds (94). In the present study, the volatile substances identified mainly included organic oxygen compounds (17, 30.91%), lipids and lipid-like molecules (15, 27.27%), organic acids and derivatives (8, 14.55%), organoheterocyclic compounds (8, 14.55%), benzenoids (6, 10.91%), and hydrocarbons (1, 1.82%; Supplementary Figure 8; Supplementary Table 4). In general, the volatile components identified in this study were similar to those previously reported, although there were some differences. This might be caused by the different experimental materials, different experimental treatments, and different sample pretreatments. In particular, we identified 27 differential volatile substances among the H, M, and S treatments, and there were 11, 9, and 7 differential volatile substances showing the highest abundance in the H, M, and S treatments, respectively (Table 2). It was inferred that different processing methods had a great impact on the volatile substances in *P. palustre*, especially the M treatment. The fermentation in the H treatment, the constant temperature (50°C) in the H treatment, and the variable temperature in the S treatment might account for the differences in volatile substances in *P. palustre*.

## 5. Conclusion

To study the effects of different processing methods on the quality, nutrition, and flavor of *Platostoma palustre*, we adopted the LC-MS and HS-GC-MS methods to compare the effects of tedding (S), sweating (M), and drying (H) on the metabolites and volatile substances of *P. palustre*. The results showed that the M treatment facilitated the stabilization and improvement of the polysaccharide and volatile substances quality of *P. palustre*, while the H treatment could promote the nutrition level of *P. palustre*. Considering the quality of polysaccharides and volatile substances, the M treatment was the optimal processing method for *P. palustre*. This study provides a theoretical reference for establishing standardized processing methods and maintaining the quality stability of *P. palustre* in future.

## Data availability statement

The original contributions presented in the study are included in the article/Supplementary material, further inquiries can be directed to the corresponding author.

## Author contributions

DT: conceptualization, methodology, data curation, formal analysis, writing—original draft, writing—reviewing and editing, and funding acquisition. FW: supervision, methodology, writing—reviewing and editing, and funding acquisition. CQ and SH: software, data curation, and formal analysis. All authors contributed to the article and approved the submitted version.
