# Supplementary material for: Integrating LC-MS and HS-GC-MS for the metabolite characterization of the Chinese medicinal plant Platostoma palustre under different processing methods
Source: Front Nutr. 2023 May 19;10:1181942. doi: 10.3389/fnut.2023.1181942 (PMC10235517; doi:10.3389/fnut.2023.1181942)
Supplement: Supplementary file 2 [file Data_Sheet_1.docx]

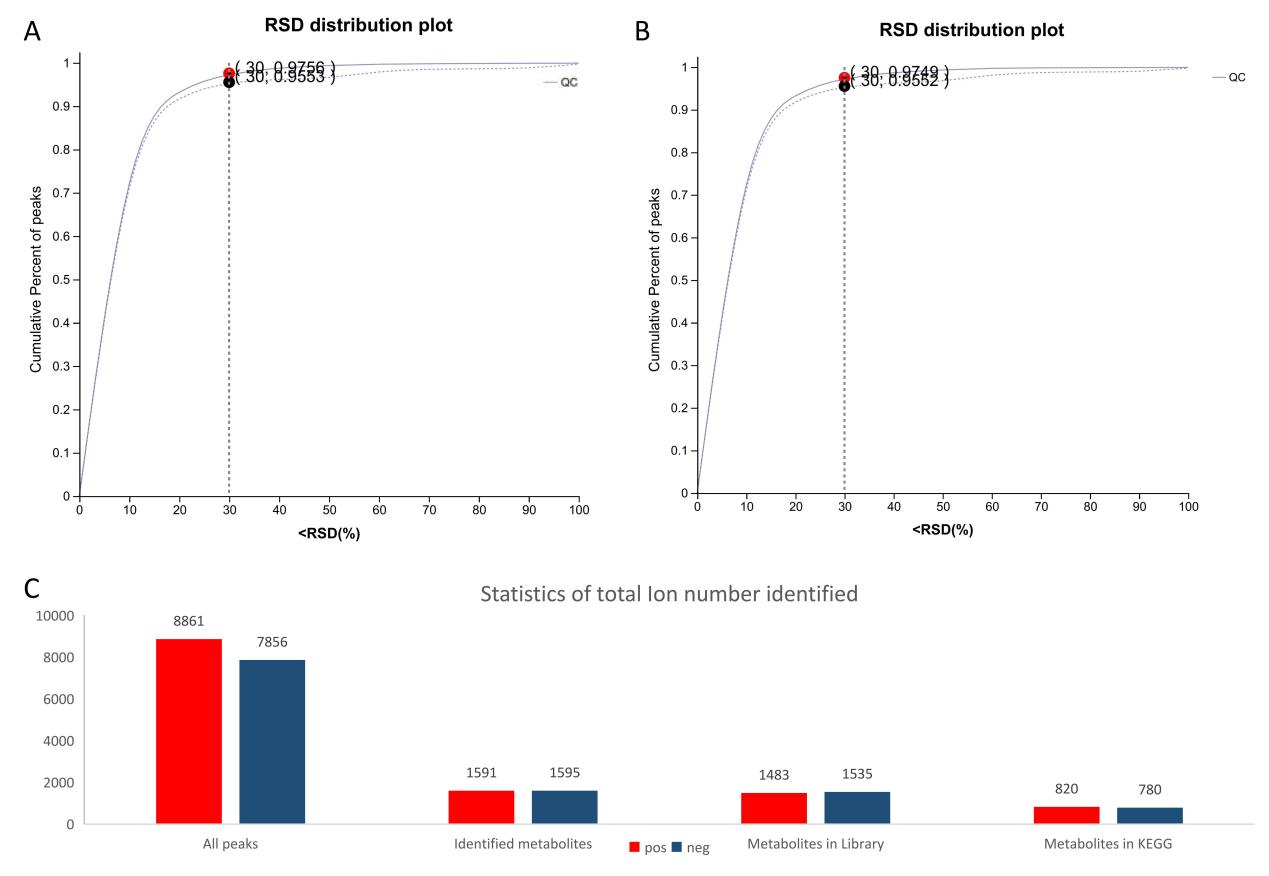


Figure S1 QC Sample Evaluation Chart and statistics of total ion number identified. A, RSD distribution plot under positive ion mode. B, RSD distribution plot under negative ion mode. C, Statistics of total ion number identified.


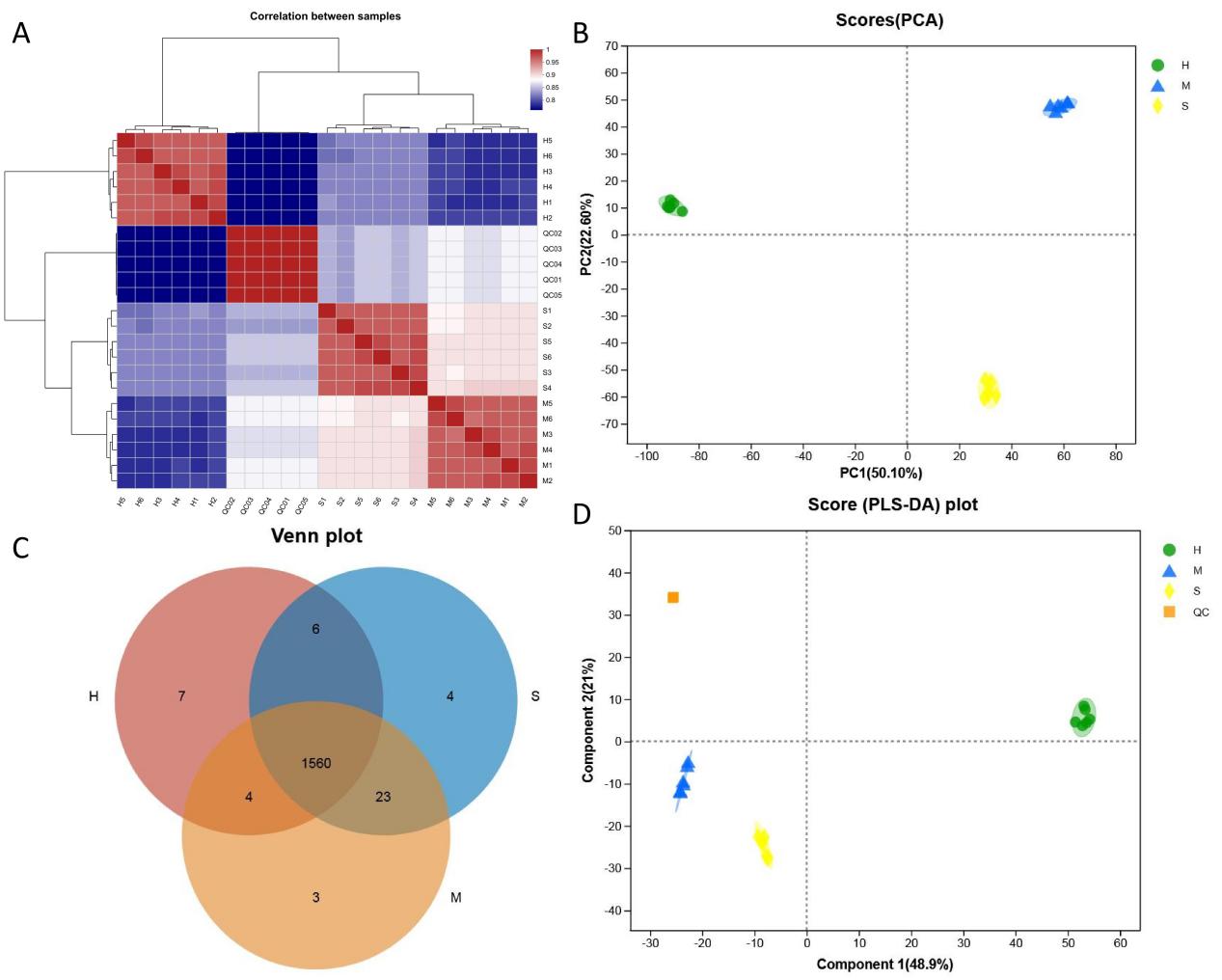


Figure S2 Comparative analysis of samples. A, Correlation between samples. B, PCA analysis. C, Venn plot. D, PLS-DA plot.


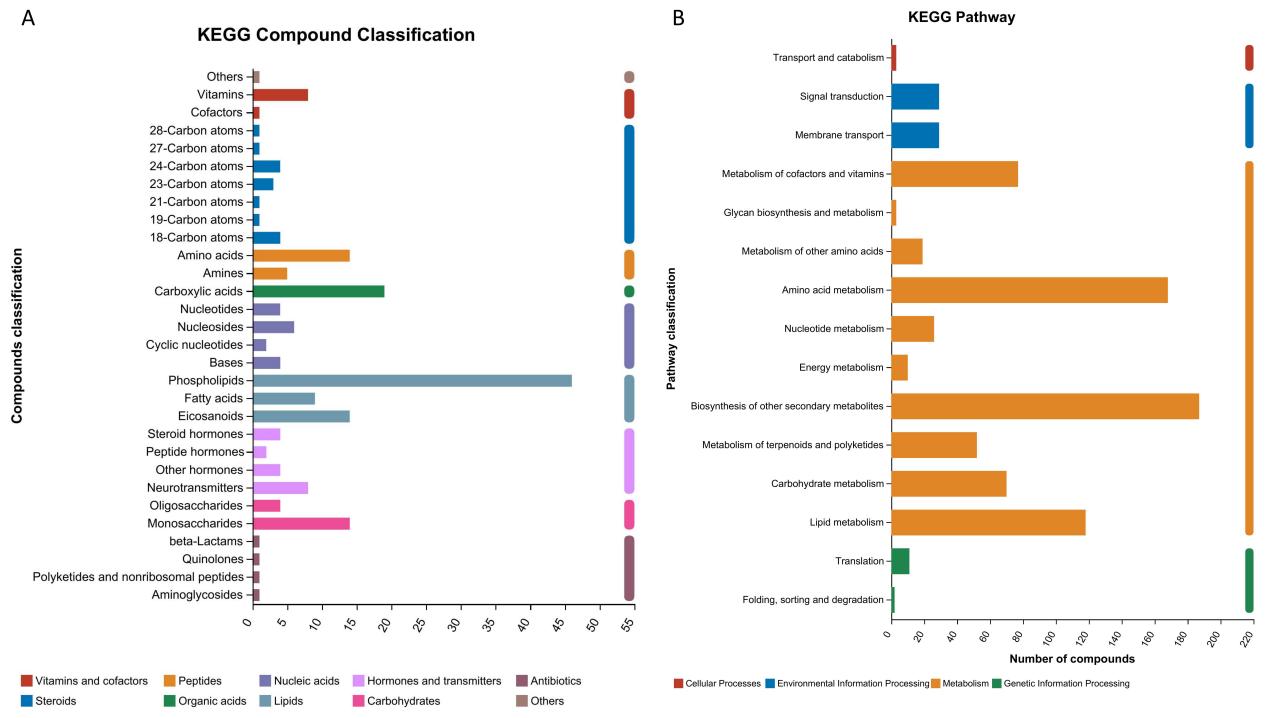


Figure S3 KEGG analysis. A, KEGG compound classification. B, KEGG pathway.


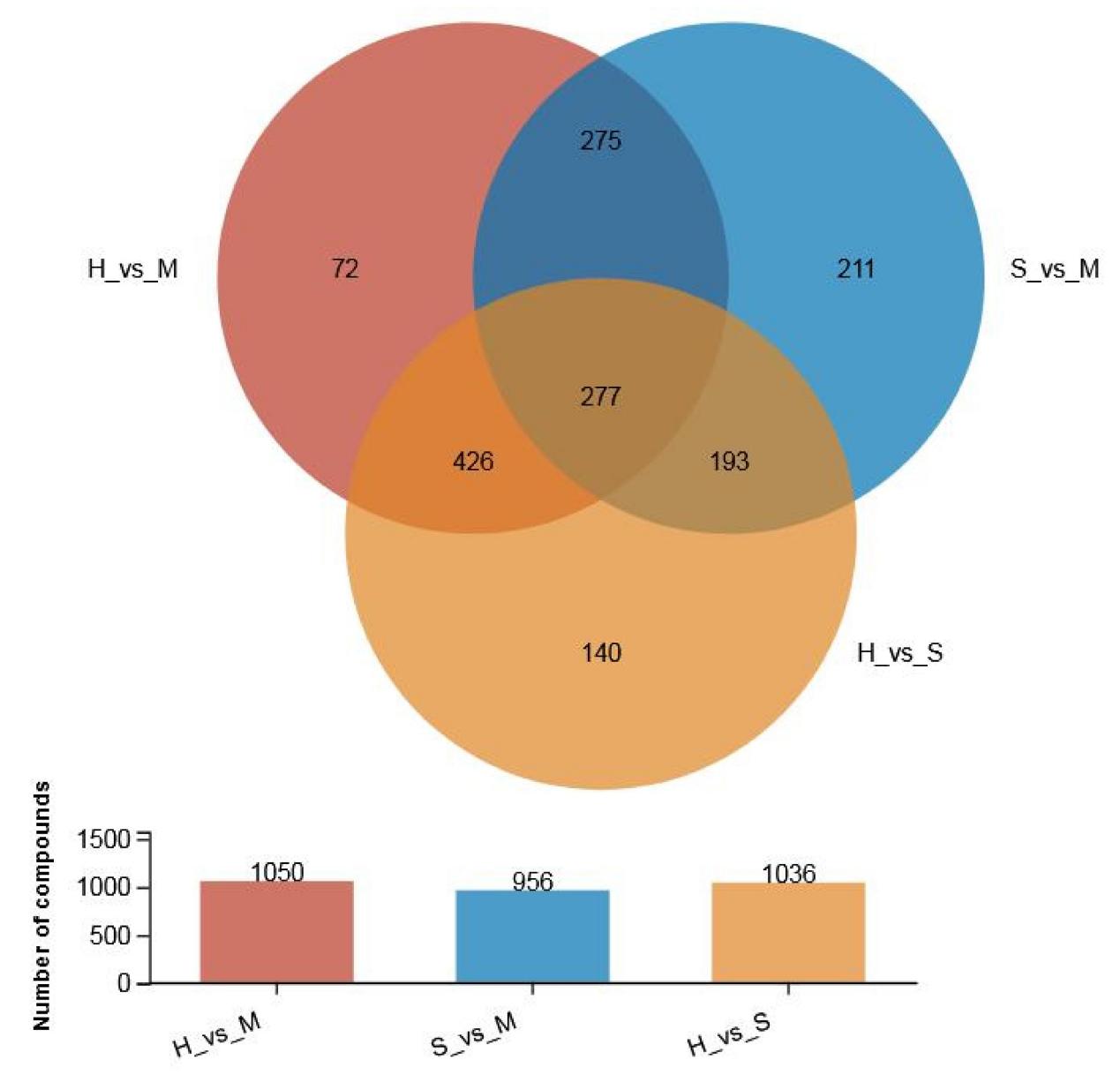


Figure S4 Venn analysis of the differential metabolites.


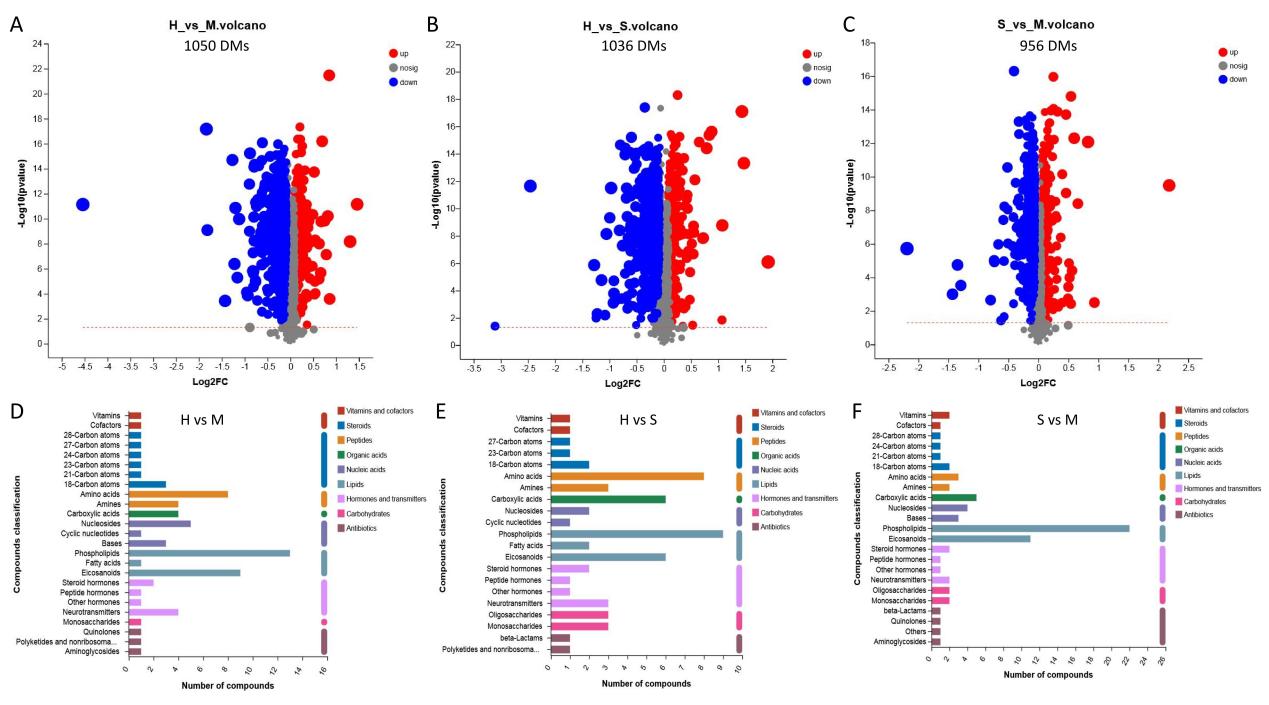


Figure S5 Volcano plot and compounds classification of different comparison groups. A-C, Volcano plot of H_vs_M, H_vs_S, and M_vs_S, respectively. D-E, Compounds classification of H_vs_M, H_vs_S, and M_vs_S, respectively.


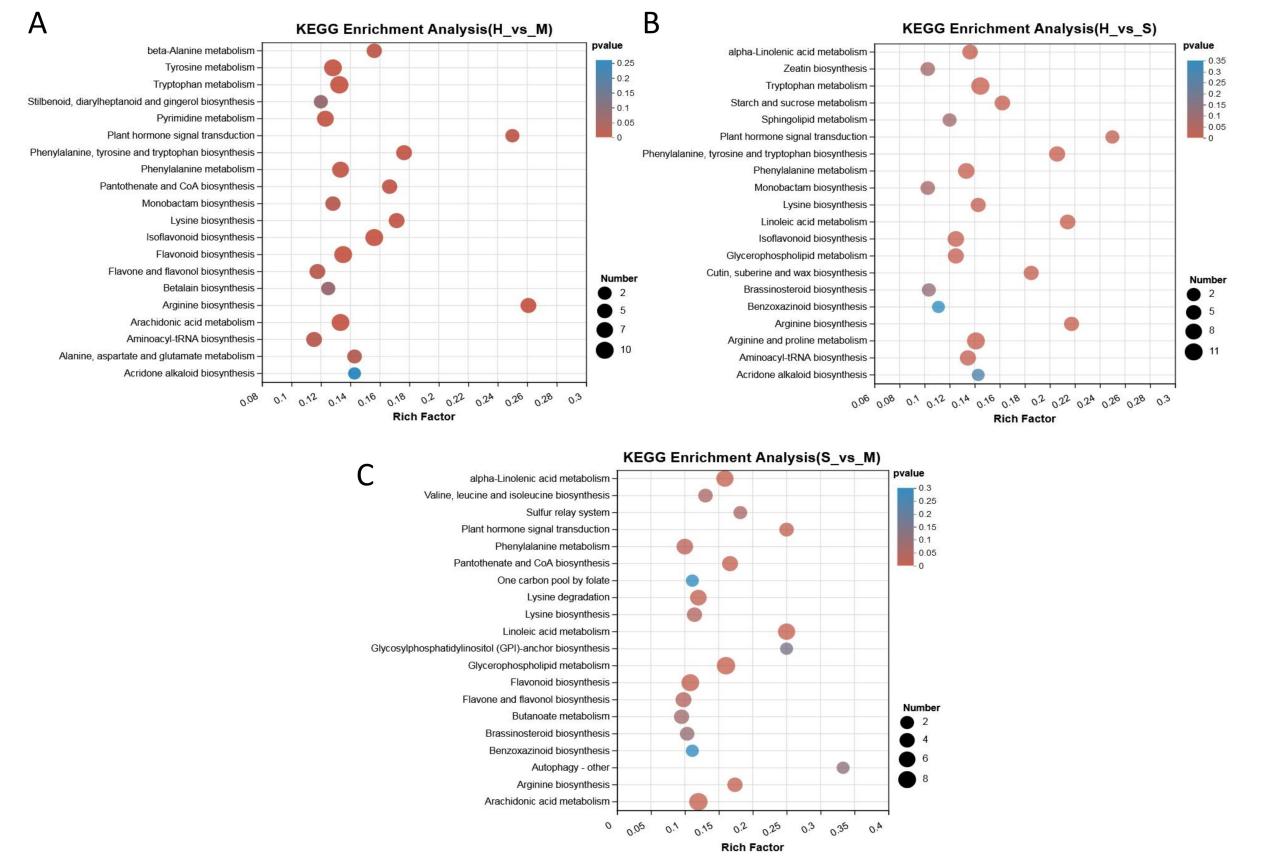


Figure S6 KEGG enrichment analysis of differential metabolites among H_vs_M, H_vs_S, and M_vs_S.


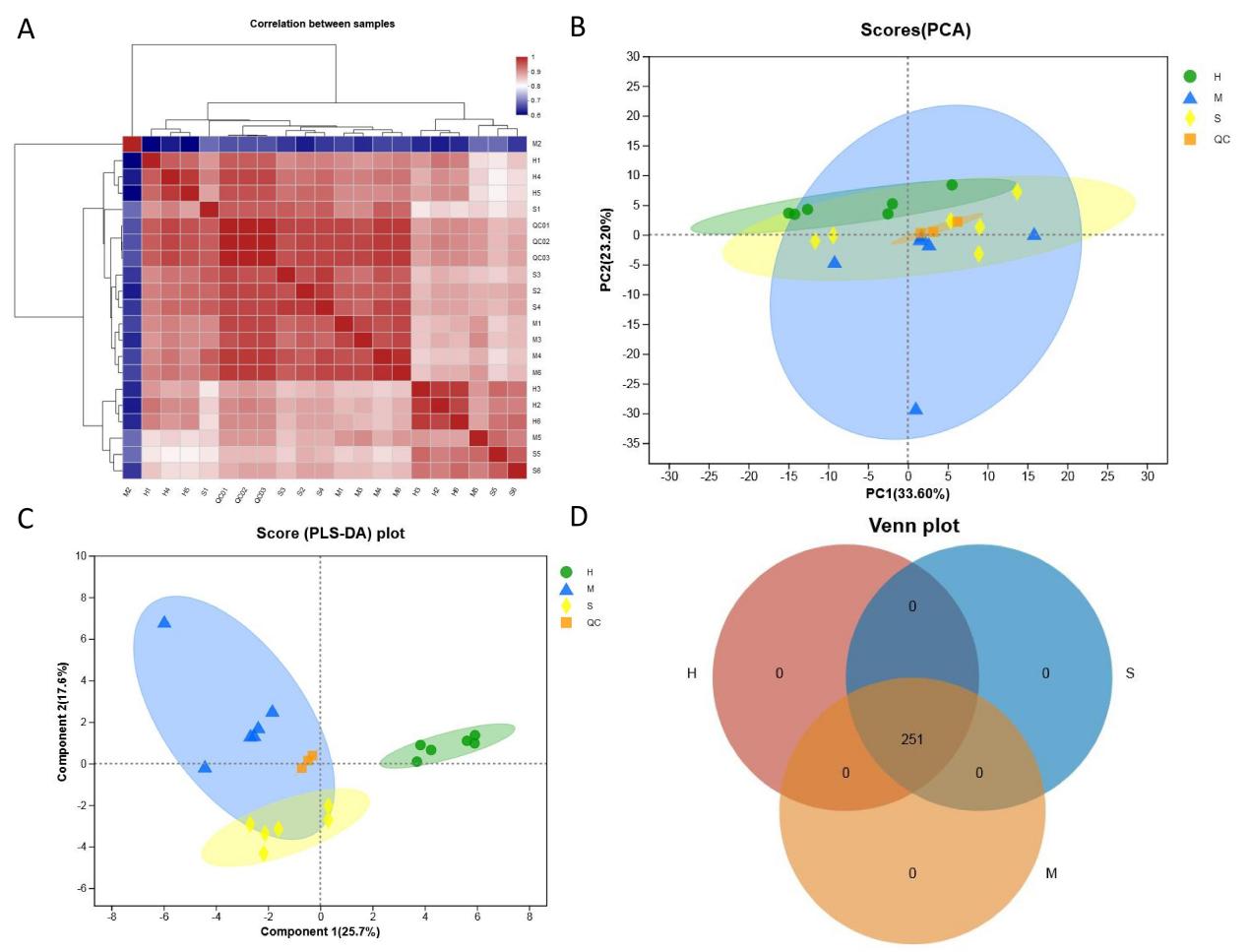


Figure S7 Comparative analysis of samples. A, Correlation between samples. B, PCA analysis. C, PLS-DA plot. D, Venn plot.


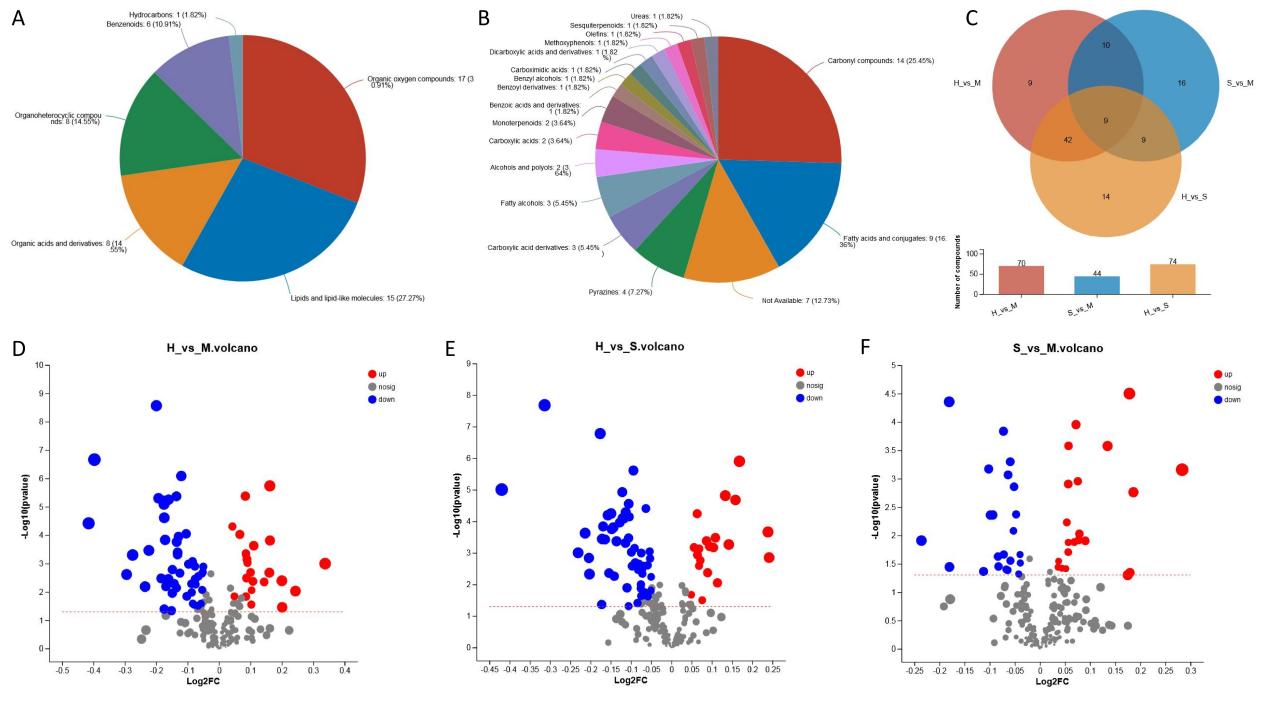


Figure S8 The classification of the volatile substances identified in this study. A, Superclass classification of the volatile substances identified in this study. B, Subclass classification of the volatile substances identified in this study. C, The differential volatile substances were detected in this study. D-E, The differential volatile substances of H_vs_M, H_vs_S, and M_vs_S, respectively.


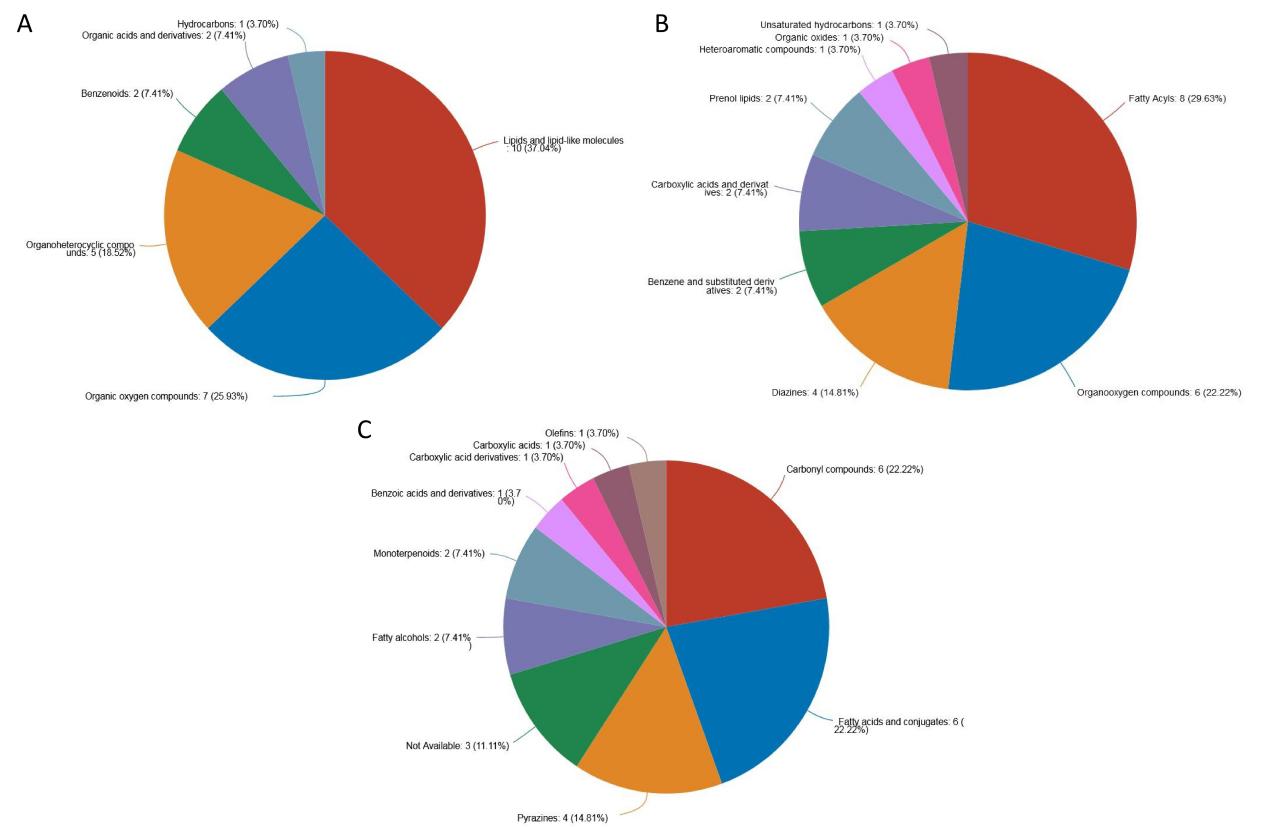


Figure S9 A-C, The superclass, class, and subclass classification of the differential volatile substances.
